# Chloroplast phosphate transporter CrPHT4-7 regulates phosphate homeostasis and photosynthesis in Chlamydomonas

**DOI:** 10.1093/plphys/kiad607

**Published:** 2023-11-14

**Authors:** Dávid Tóth, Soujanya Kuntam, Áron Ferenczi, André Vidal-Meireles, László Kovács, Lianyong Wang, Zsuzsa Sarkadi, Ede Migh, Klára Szentmihályi, Roland Tengölics, Juliane Neupert, Ralph Bock, Martin C Jonikas, Attila Molnar, Szilvia Z Tóth

**Affiliations:** Institute of Plant Biology, HUN-REN Biological Research Centre, H-6726 Szeged, Hungary; Doctoral School of Biology, University of Szeged, H-6722 Szeged, Hungary; Institute of Plant Biology, HUN-REN Biological Research Centre, H-6726 Szeged, Hungary; Institute of Molecular Plant Sciences, School of Biological Sciences, King's Buildings, University of Edinburgh, Edinburgh EH9 3BF, UK; Institute of Plant Biology, HUN-REN Biological Research Centre, H-6726 Szeged, Hungary; Institute of Plant Biology, HUN-REN Biological Research Centre, H-6726 Szeged, Hungary; Department of Molecular Biology, Princeton University, Lewis Thomas Laboratory, Princeton, NJ 08544, USA; Institute of Biochemistry, HUN-REN Biological Research Centre, H-6726 Szeged, Hungary; Hungarian Centre of Excellence for Molecular Medicine—Biological Research Centre Metabolic Systems Biology Research Group, H-6726 Szeged, Hungary; Institute of Biochemistry, HUN-REN Biological Research Centre, H-6726 Szeged, Hungary; Institute of Materials and Environmental Chemistry, Research Centre for Natural Sciences, H-1117 Budapest, Hungary; Hungarian Centre of Excellence for Molecular Medicine—Biological Research Centre Metabolic Systems Biology Research Group, H-6726 Szeged, Hungary; Metabolomics Lab, Core Facilities, HUN-REN Biological Research Centre, H-6726 Szeged, Hungary; Max Planck Institute of Molecular Plant Physiology, D-14476 Potsdam-Golm, Germany; Max Planck Institute of Molecular Plant Physiology, D-14476 Potsdam-Golm, Germany; Department of Molecular Biology, Princeton University, Lewis Thomas Laboratory, Princeton, NJ 08544, USA; Howard Hughes Medical Institute, Princeton University, Lewis Thomas Laboratory, Princeton, NJ 08544, USA; Institute of Molecular Plant Sciences, School of Biological Sciences, King's Buildings, University of Edinburgh, Edinburgh EH9 3BF, UK; Institute of Plant Biology, HUN-REN Biological Research Centre, H-6726 Szeged, Hungary

## Abstract

In eukaryotic cells, phosphorus is assimilated and utilized primarily as phosphate (Pi). Pi homeostasis is mediated by transporters that have not yet been adequately characterized in green algae. This study reports on PHOSPHATE TRANSPORTER 4-7 (CrPHT4-7) from *Chlamydomonas reinhardtii*, a member of the PHT4 transporter family, which exhibits remarkable similarity to AtPHT4;4 from Arabidopsis (*Arabidopsis thaliana*), a chloroplastic ascorbate transporter. Using fluorescent protein tagging, we show that CrPHT4-7 resides in the chloroplast envelope membrane. *Crpht4-7* mutants, generated by the CRISPR/Cas12a-mediated single-strand templated repair, show retarded growth, especially in high light, reduced ATP level, strong ascorbate accumulation, and diminished non-photochemical quenching in high light. On the other hand, total cellular phosphorous content was unaffected, and the phenotype of the *Crpht4-7* mutants could not be alleviated by ample Pi supply. *CrPHT4-7*-overexpressing lines exhibit enhanced biomass accumulation under high light conditions in comparison with the wild-type strain. Expressing CrPHT4-7 in a yeast (*Saccharomyces cerevisiae*) strain lacking Pi transporters substantially recovered its slow growth phenotype, demonstrating that CrPHT4-7 transports Pi. Even though CrPHT4-7 shows a high degree of similarity to AtPHT4;4, it does not display any substantial ascorbate transport activity in yeast or intact algal cells. Thus, the results demonstrate that CrPHT4-7 functions as a chloroplastic Pi transporter essential for maintaining Pi homeostasis and photosynthesis in *C. reinhardtii*.

## Teaser

A *Chlamydomonas reinhardtii* phosphate transporter localizes in the chloroplast envelope membrane and contributes to maintaining phosphate homeostasis and photosynthesis.

## Introduction

Phosphorus is a vital element for all living organisms and is present in every compartment of the plant cell. It is an integral component of proteins, sugar phosphates, nucleic acids, and structural phospholipids, and is also necessary for information transfer via signal cascades (Dyhrman 2016).

Plants absorb phosphorus from the soil in the form of inorganic phosphate (Pi) through the cell wall and plasma membrane. This Pi is then transported to various cell organelles. Despite its ubiquitous presence in the environment, Pi availability often restricts plant growth due to phosphate complexation with metal cations and organic particles in the soil (e.g. Gutiérrez-Alanís et al. 2018, Crombez et al. 2019). Phosphorus starvation severely impacts cellular metabolism, leading to slowed growth, alterations in protein, lipid and starch biosynthesis and degradation, changes in cellular respiration, and recycling of internal structures and compounds (reviewed by Sanz-Luque and Grossman 2023). Hence, efficient acquisition and storage of phosphorus, as well as the ability to adapt to phosphorus limitation, are critical factors that determine plant productivity.

Fertilizers, sourced from nonrenewable rock phosphate, enhance crop yields which would otherwise be constrained by the availability of Pi. However, the leaching of surplus Pi into aquatic ecosystems leads to environmental issues such as eutrophication. Given these factors, the study of Pi uptake and transport in plants is of high importance.

PHOSPHATE TRANSPORTER (PHT) family members are the best-studied phosphate transporters in vascular plants. They are well known for their roles in Pi uptake from soil and Pi translocation within the plant (Versaw and Garcia 2017; Wang et al. 2021). Arabidopsis (*Arabidopsis thaliana*) has 5 high-affinity Pi transporter families (PHT1-5) that are distinguished based on their functional differences and subcellular localization. The PHT1 proteins are plasma membrane proton-coupled Pi-symporters that mediate Pi acquisition from the soil and Pi translocation within the plant. Members of the PHT2 and PHT4 families are present in plastids and in the Golgi apparatus, whereas PHT3 transporters are found in mitochondria and PHT5;1 is a vacuolar Pi transporter (Versaw and Garcia 2017; Srivastava et al. 2018).

Phosphate transport is poorly studied in green algae and surprisingly, no Pi transporter has been characterized in detail (Wang et al. 2020). Understanding the mechanisms of Pi uptake and cellular distribution is highly relevant since microalgae can accumulate and store large amounts of Pi in the form of polyphosphate granules in specific vacuoles called acidocalcisomes (Sanz-Luque et al. 2020). This so-called “luxury uptake” (Riegman et al. 2000) may enable recovery of Pi upon wastewater treatment (Shilton et al. 2012) to subsequently produce phosphate-rich fertilizers (Slocombe et al. 2020). Thus, understanding Pi uptake and transport in microalgae are of high importance to the protection of the environment and water management.

The *PHT* gene family in *Chlamydomonas reinhardtii* contains 25 putative *PHT* genes, categorized in 4 subfamilies, namely *PHOSPHATE TRANSPORTER A* (*CrPTA*), *PHOSPHATE TRANSPORTER B* (*CrPTB*), *CrPHT3*, and *CrPHT4* (Wang et al. 2020). The *CrPTA*, *CrPTB*, *CrPHT3*, and *CrPHT4* subfamilies may contain 4, 11, 1, and 9 members, respectively (Wang et al 2020). Members of the CrPTA family, a sister family of PHT1 in land plants, may be found in the plasma membrane (Wang et al. 2020) or targeted to secretory and other pathways (Tardif et al. 2012, Wang et al. 2023). CrPTB members were shown or predicted to be targeted to the secretory and other pathways (Tardif et al. 2012, Wang et al. 2020, 2023); however, based on homology with streptophyte algae, they are likely to be located in the plasma membrane (Bonnot et al. 2017). CrPHT3 (Cre03.g172300) is possibly found in mitochondria (Tardif et al. 2012, Wang et al. 2020, 2023). Several CrPHT4 family members are predicted to be localized in the chloroplast, whereas others may be targeted to secretory pathways or the mitochondria (Tardif et al. 2012, Wang et al. 2020, 2023). It is interesting to note that *CrPHT* transcript levels responded differently to Pi starvation, with most genes belonging to the *CrPTA* and *CrPTB* families showing remarkable inductions (Moseley et al. 2006, Wang et al. 2020).

Here, we investigated a member of the CrPHT4 family, called CrPHT4-7 (Cre16.g663600, called CrPHT7 in Phytozome v. 13). This transporter has several PHT4 homologs in Arabidopsis with varied location and roles: *AtPHT4;1* to *AtPHT4;5* are expressed in plastids, whereas *AtPHT4;6* in the Golgi apparatus (Guo et al. 2008a; reviewed by Versaw and Garcia 2017, Fabiańska et al. 2019). AtPHT4;1 was found in the thylakoid membranes (Pavón et al. 2008), and AtPHT4;4 in the chloroplast envelope membrane of mesophyll cells (Miyaji et al. 2015). The expressions of *AtPHT4;3* and *AtPHT4;5* are restricted mostly to leaf phloem cells, and *AtPHT4;2* is most highly expressed in the roots and other non-photosynthetic tissues (Guo et al. 2008b). All AtPHT4 transporters may act as phosphate transporter as they could complement the yeast PAM2 mutant lacking Pi transporters (Guo et al. 2008a), and they exhibit H^+^ and/or Na^+^-coupled Pi transport activities (Guo et al. 2008a, Irigoyen et al. 2011, Miyaji et al. 2015). Interestingly, it was found that AtPHT4;4 transports ascorbate (Asc) into the chloroplasts (Miyaji et al. 2015), to ensure appropriate Asc level for its multiple roles (Tóth 2023). AtPHT4;1, on the other hand, may export Pi out of the thylakoid lumen (Karlsson et al. 2015). AtPHT4;2 has been shown to act bidirectionally, and its suggested physiological role is to export Pi from root plastids to support ATP homeostasis (Irigoyen et al. 2011).

We discovered that CrPHT4-7 is a Pi transporter located in the chloroplast envelope membrane of *C. reinhardtii*, and it is required for maintaining Pi homeostasis and optimal photosynthesis under high light conditions.

## Results

### CrPHT4-7 is localized in the chloroplast envelope membrane

CrPHT4-7 belongs to the PHT4 family of transporters, showing similarity to members of the solute carrier family 17 (sodium-dependent Pi co-transporter, SLC17A). CrPHT4-7 shows 42.6% similarity to the *A. thaliana* AtPHT4;5 (AT5G20380) Pi transporter and around 29% to 36% similarity with other Pi transporters in the PHT4 family, namely AtPHT4;2, 4;1, 4;6, and 4;3. CrPHT4-7 also shows a relatively high, 37.4% similarity to the chloroplastic Asc transporter AtPHT4;4 (AT4G00370.1) (according to Phytozome v.13, see Supplemental Fig. S1 for the sequence alignments).

In *Arabidopsis*, AtPHT4 transporters are located in the chloroplast envelope membrane of plastids, in thylakoid membranes, and in the Golgi apparatus (reviewed by Fabiańska et al. 2019). Prediction algorithms do not provide a clear indication as to where CrPHT4-7 is localized within the cell. According to DeepLoc 1.0 (Thumuluri et al. 2022), CrPHT4-7 is associated with the Golgi apparatus, whereas LocTree 3 (Goldberg et al. 2014) predicts that the mature protein is localized in the chloroplast membrane. In contrast, ChloroP 1.1 (Emanuelsson et al. 1999) indicates that CrPHT4-7 is not targeted to the chloroplast, and PredAlgo 1.0 (Tardif et al. 2012) predicts that is not in the chloroplast, mitochondria or secretory pathway. The in silico analysis by Wang et al. (2020) suggested that CrPHT4-7 is likely localized in the secretory pathway. The recently developed protein prediction tool PB-Chlamy predicts that PHT4-7 is found in the chloroplast (Wang et al. 2023).

**Figure 1.**
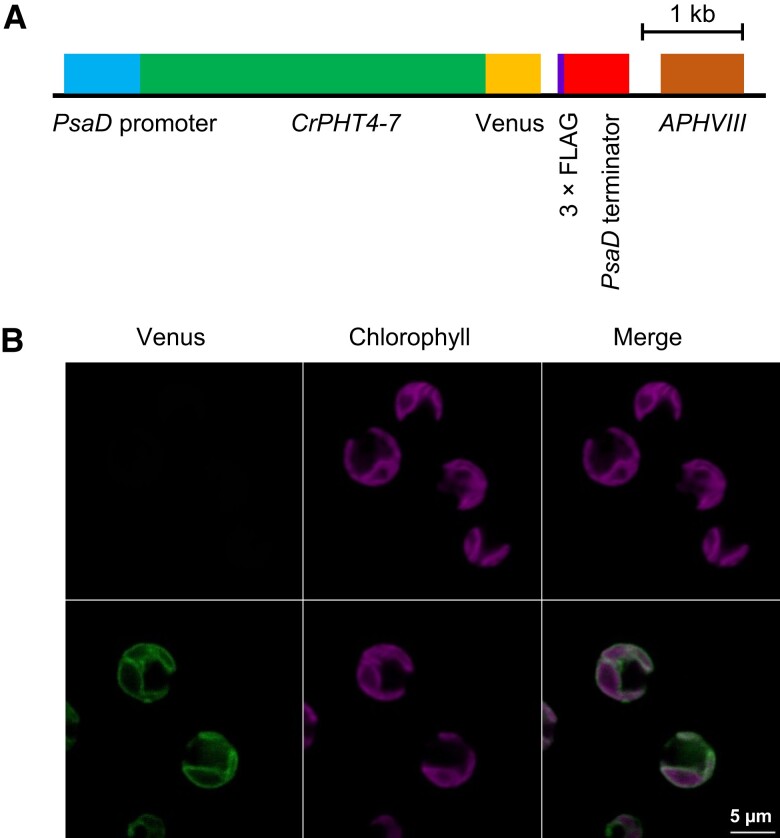
CrPHT4-7 is found in the chloroplast envelope membrane. **A)** Map of the pLM005-CrPHT4-7 plasmid expressing a Venus-tagged CrPHT4-7 version. **B)** Representative fluorescence microscopic images of the UVM11 strain (upper row) and the UVM11 strain expressing pLM005-CrPHT4-7 with Venus-3×FLAG (lower row). Venus fluorescence and Chl auto-fluorescence were detected between 520 and 540 nm and 650 and 750 nm, respectively. The merged Venus + Chl fluorescence image is also shown. Scale bar: 5 *μ*m, applicable to all images.

To determine the subcellular location of CrPHT4-7, we tagged the full-length *CrPHT4-7* gene including the introns with the fluorescent marker Venus (Nagai et al. 2002) at the C-terminus. The resulting construct (pLM005-*CrPHT4-7*, Fig. 1A) was then introduced the into the UVM11 strain that has been shown to support enhanced transgene expression (Neupert et al. 2009, 2020). In parallel, we also introduced the construct into the *Chlamydomonas* CC-4533 strain (also called cMJ030), which is the host strain used in the Chlamydomonas Library Project (Fauser et al. 2022, Wang et al. 2023). In both the UVM11 and CC-4533 strains, the fluorescent signals from Venus-tagged CrPHT4-7 could be detected (Fig. 1, and Supplemental Fig. S2, respectively). In the case of the UVM11 strain, the signal was detected in 41 out of 93 transformed clones tested (corresponding to 44% efficiency). The merged images of Venus-tagged CrPHT4-7 and Chl *a* auto-fluorescence (Fig. 1, Supplemental Fig. S2) show that CrPHT4-7 is localized to the chloroplast envelope.

### CrPHT4-7 is required for normal growth especially at high light

To investigate the physiological role of CrPHT4-7, we studied *pht4-7* knock out mutants, which were generated by CRISPR/Cas12a-mediated single-strand templated repair introducing early stop codons (Ferenczi et al. 2017). In the initial CRISPR/Cas12a-ssODN mutagenesis screen, the *pht4-7* mutants formed smaller colonies than wild-type (WT, CC-1883) cells (Ferenczi et al. 2017). In agreement with this observation, 5 independent mutant lines showed a similar slow growth phenotype in comparison with the WT strain as estimated by absorbance at 720 nm (OD_720_) in a Multi-Cultivator photobioreactor (Supplemental Fig. S3; Thoré et al. 2021). Of these, we have randomly selected 2 independent mutants, called *pht4-7#7* and *#9* for further detailed analyses.

**Figure 2.**
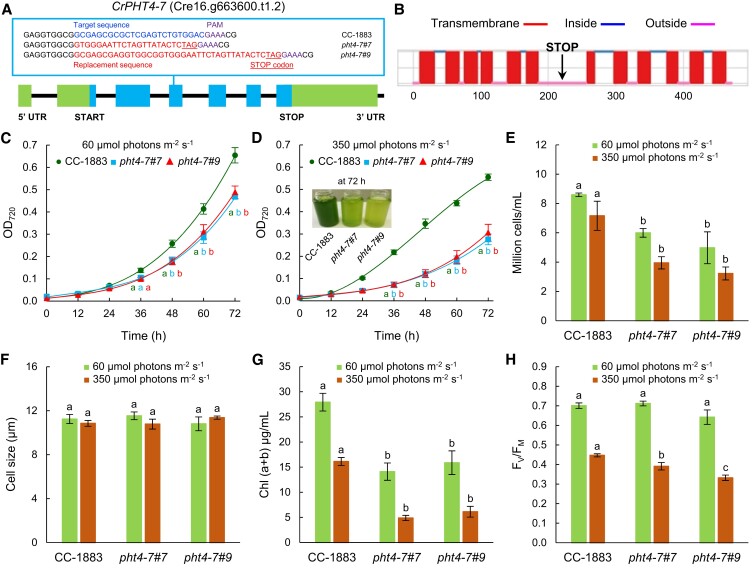
*pht4-7* mutants generated via the CRISPR/Cas12a technique exhibit diminished fitness. **A)** Physical map of *CrPHT4-7* (obtained from Phytozome, v. 13) with the replacement sequence including a stop codon, and a PAM sequence in the third exon in the *Crpht4-7#7 and #9* mutants. Exons are shown as blue boxes, introns as black lines, and promoter/5′ UTR and terminator sequences as green boxes. **B)** Prediction of transmembrane helices of CrPHT4-7 by Deep TMHMM v. 1.0.24. The introduction of the stop codon prevents the translation of at least 6 transmembrane helices. **C)** Culture growth of *pht4-7* mutants and the CC-1883 wild type, in TAP medium in continuous illumination of 60 *µ*mol photons m^−2^ s^−1^ at 23 °C, bubbled with air for 72 h in a Multi-Cultivator photobioreactor. The initial Chl content was set to 0.5 *µ*g Chl(a + b)/mL. **D)** Culture growth in TAP medium under continuous illumination of 350 *µ*mol photons m^−2^ s^−1^ at 23 °C, bubbled with air for 72 h in a Multi-Cultivator photobioreactor. The initial Chl content was set to 0.5 *µ*g Chl(a + b)/mL. A photograph of an aliquot of the cultures after 72 h of growth is shown in the inset. **E)** Cell numbers at 60 and 350 *µ*mol photons m^−2^ s^−1^ after 72 h of growth. **F)** Cell sizes at 60 and 350 *µ*mol photons m^−2^ s^−1^. **G)** Chl(a + b) contents after 72 h of growth at 60 and 350 *µ*mol photons m^−2^ s^−1^ in a photobioreactor. **H)***F_V_*/*F_M_* values after 72 h of growth at 60 and 350 *µ*mol photons m^−2^ s^−1^. The averages and standard errors presented in panels **C)** to **F)** are based on 3 to 5 independent experiments with 2 to 6 biological replicates in each. The significance of differences between means was determined by ANOVA with Tukey post hoc test. The means with different letters are significantly different (*P* < 0.05).

The presence of the introduced sequence variations and premature stop codons was confirmed by Sanger sequencing in the *pht4-7#7* and *#9* mutant lines (Fig. 2A). The stop codons were introduced into the third exon of *CrPHT4-7* to prevent the translation of half of the C-terminal transmembrane helices (Fig. 2B). Consequently, the *pht4-7#7* and *#9* mutants are very likely to express a strongly truncated, nonfunctional form of CrPHT4-7.

In agreement with the preliminary experiments, a significant difference in biomass accumulation (as assessed by OD_720_) between the WT and *pht4-7* mutant lines was found when grown at normal light (60 *µ*mol photons m^−2^ s^−1^, measured inside the culture tube; Fig. 2C). At high light (350 *µ*mol photons m^−2^ s^−1^), the fitness penalty associated with the absence of *pht4-7* became even more pronounced (Fig. 2D). Accordingly, the cell number and the Chl concentrations of the cultures (Chl(a + b)/mL) measured after 3 d of growth were significantly lower in the mutants than in the WT at both 60 and 350 *µ*mol photons m^−2^ s^−1^ (Figs. 2, E and G). We noted that the cell sizes of the mutants and the WT were very similar at normal and high light (Fig. 2F).

The *F_V_*/*F_M_* value, an indicator of photosynthetic performance (Schansker et al. 2014, Sipka et al. 2021), was approximately 0.65 to 0.7 in all genotypes at normal light (Fig. 2H), which is typical for *C. reinhardtii* (e.g. Bonente et al. 2012, Santabarbara et al. 2019). At intense illumination, the *F_V_*/*F_M_* value was about 0.45 in the WT, indicating downregulation of photosynthetic electron transport possibly involving photoinhibition. The reduction of photosynthetic efficiency was more enhanced in the *pht4-7* mutants than in the WT strain (Fig. 2H). From the above data, we conclude that CrPHT4-7 is required for cellular fitness, particularly under intense illumination.

Measurements were also conducted on cultures cultivated under photoautotrophic conditions, specifically in a high salt (HS) medium, with normal light and CO_2_ supplementation. The observed growth rate of these cultures was notably slower compared to those in TAP medium (Supplemental Fig. S4 vs. Fig. 2C). This slower growth rate was consistent across both the WT and the *pht4-7* mutants, with no statistically significant differences between them. Consequently, it can be inferred that photoautotrophic conditions appeared to mitigate rather than exacerbate the growth phenotype of the *pht4-7* mutants (Supplemental Fig. S4).

### Is CrPHT4-7 an ascorbate or a phosphate transporter?

Since CrPHT4-7 shows high amino acid sequence similarity to the AtPHT4;4 Asc transporter, we decided to assess Asc metabolism and function. This analysis and the consecutive ones were carried out on alga cultures grown in TAP medium in Erlenmeyer flasks, enabling cultivating many more cultures in parallel than in the Multi-Cultivator instrument. By determining the cell number and the Chl content of the cultures after 3 d of growth in the Erlenmeyer flasks, we could confirm that the *pht4-7* mutant cultures grow more slowly than the WT especially at high light (Supplemental Fig. S5). In comparison with the Multi-Cultivator instrument, the difference between the mutants and WT was milder, indicating that shake-flask culturing was less stressful for the cells than growing in the Multi-Cultivator (see also Materials and methods section).

**Figure 3.**
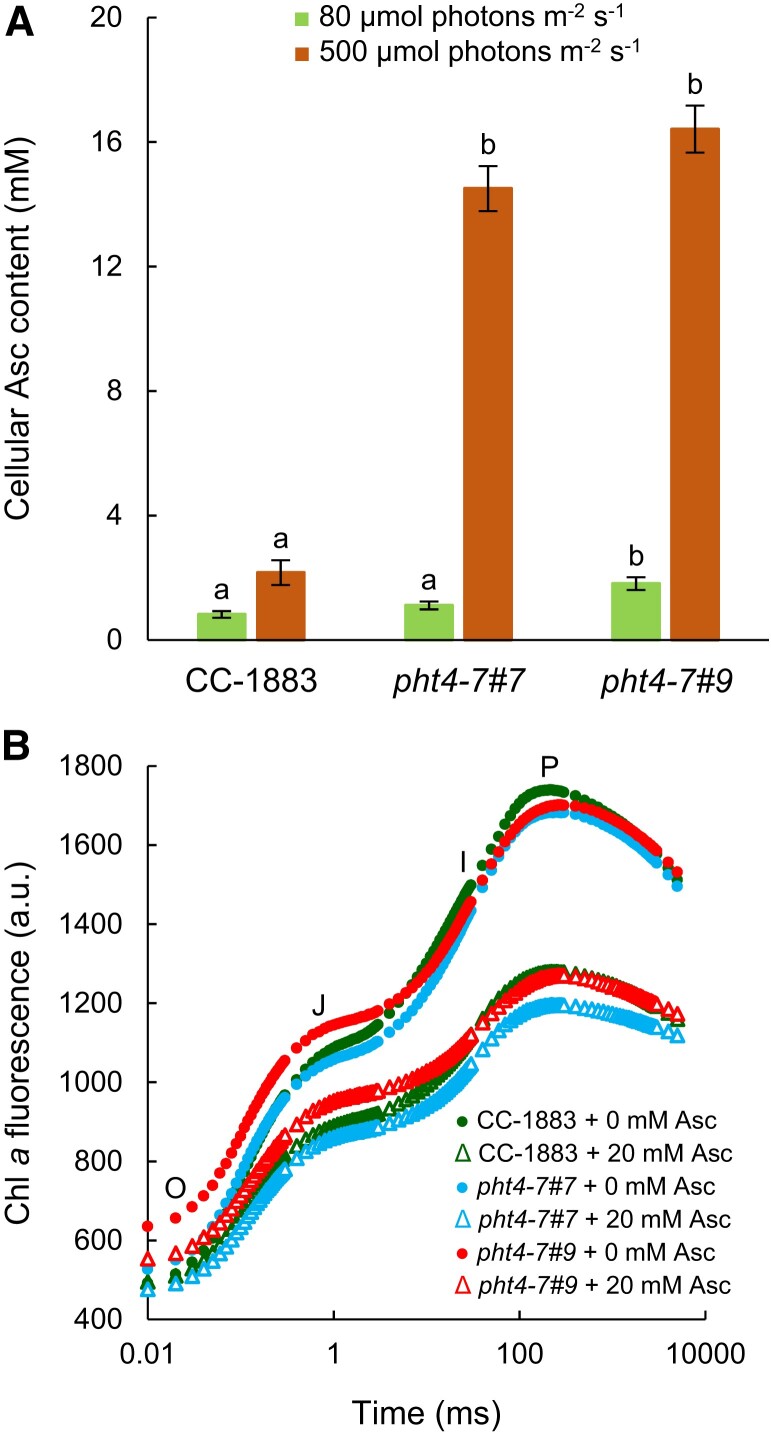
The *pht4-7* mutation leads to strong ascorbate (Asc) accumulation at high light and does not affect chloroplastic Asc uptake. **A)** Asc content of the *pht4-7* mutants and the CC-1883 strain after 72 h of growth in TAP medium at 80 and 500 *µ*mol photons m^−2^ s^−1^. **B)** Fast Chl *a* fluorescence (OJIP) transients measured with or without 20 mm of Asc on cultures grown at 80 *µ*mol photons m^−2^ s^−1^. The cultures were grown in Erlenmeyer flasks. The averages and standard errors are based on 3 to 6 independent experiments with 2 to 4 biological replicates in each. The significance of differences between means was determined by ANOVA with Tukey post hoc test. The means with different letters are significantly different (*P* < 0.05).

The cellular Asc concentration was about 0.8 mm in the CC-1883 strain when grown at 80 *µ*mol photons m^−2^ s^−1^ (Fig. 3A), that is in the same range as in other *C. reinhardtii* WT strains (Vidal-Meireles et al. 2017, 2020). In the *pht4-7 #7* mutant, the Asc concentration was about 1.1 mm and in the *pht4-7#9* line, it was about 1.8 mm. At 500 *µ*mol photons m^−2^ s^−1^, the Asc content increased 3-fold in the WT, whereas an about a 10-fold increase was observed in both *pht4-7* mutants, reaching approximately 15 mm Asc in the cell (Fig. 3A).

Next, we investigated the effect of Asc treatment on the fast Chl *a* fluorescence kinetics, which is a sensitive method to detect alterations in the function of the photosynthetic electron transport chain (e.g. Schansker et al. 2014). It was demonstrated earlier that a 10 mm Asc treatment causes a substantial, approx. 20-fold increase in cellular Asc content; at this high concentration, Asc may inactivate the oxygen-evolving complex (OEC) in *C. reinhardtii* resulting in diminished variable Chl *a* fluorescence (Nagy et al. 2016, 2018). We hypothesized that, if CrPHT4-7 is an Asc transporter in the chloroplast envelope membrane, then Asc transport into the chloroplast would be less efficient in its absence and consequently, less damage to the OEC should occur upon Asc treatment. As anticipated, the treatment with 20 mm Asc led to a decrease in variable fluorescence in cultures grown under normal light conditions. However, no discernible differences were observed between the WT and the *pht4-7* mutants (Fig. 3B). This result indicates that CrPHT4-7 does not contribute substantially to Asc transport into the chloroplast.

**Figure 4.**
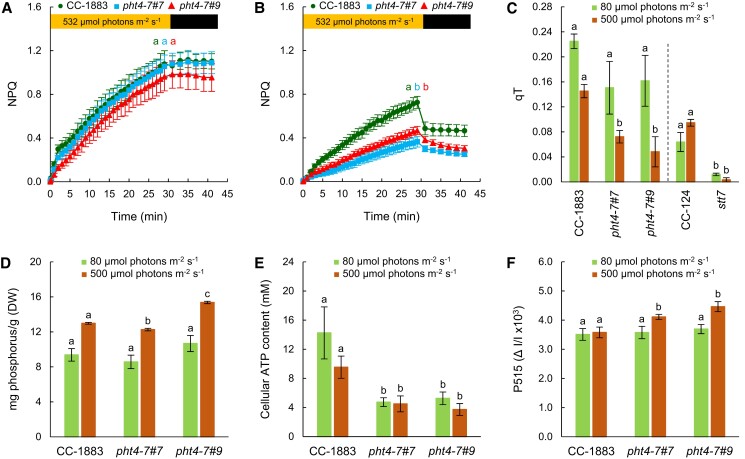
The *pht4-7* mutation alters photosynthetic redox homeostasis. **A)** NPQ of cultures grown in TAP medium at 80 *µ*mol photons m^−2^ s^−1^. **B)** NPQ of cultures grown in TAP medium at 500 *µ*mol photons m^−2^ s^−1^. For NPQ induction in panels **A)** and **B)**, light adaptation consisted of 30 min illumination at 532 *µ*mol photons m^−2^ s^−1^, followed by 12 min of dark adaptation interrupted with saturating pulses of 3,000 *µ*mol photons m^−2^ s^−1^. **C)** State transition (qT, see details in the Materials and methods section and typical kinetics in Supplemental Fig. S6). **D)** Total phosphorous content. **E)** Cellular ATP content. **F)** Total proton motive force, determined based on the absorbance change at 515 nm against the 535 nm reference wavelength, expressed in ΔI/I units. All the cultures were grown in Erlenmeyer flasks. The averages and standard errors are based on 3 to 12 independent experiments with 1 to 2 biological replicates in each. The significance of differences between means was determined by ANOVA with Tukey post hoc test. The means with different letters are significantly different (*P* < 0.05). In the cases of panels **A)** and **B)**, significance was calculated at the end of the illumination period. In panel **C)**, each mutant was compared to its own wild type. DW, dry weight.

Ascorbate is a reductant for violaxanthin deepoxidase in vascular plants (Saga et al. 2010; Hallin et al. 2016), but is not required for green algal-type violaxanthin deepoxidases (Li et al. 2016; Vidal-Meireles et al. 2020). Instead, Asc mitigates an oxidative stress-related qI component of non-photochemical quenching (NPQ) and, therefore, NPQ is increased upon Asc-deficiency in *C. reinhardtii* (Vidal-Meireles et al. 2020). As expected, when the cultures were grown in normal light in TAP medium, the rapidly developing energy-dependent phase (qE) of NPQ was basically absent and NPQ mostly consisted of a slow phase, involving the zeaxanthin-dependent (qZ), state transition (qT), and photoinhibitory (qI) components (e.g. Xue et al. 2015; Vidal-Meireles et al. 2020). The NPQ kinetics of the *pht4-7* mutants and the WT were similar in normal light (Fig. 4A). In high light, NPQ diminished remarkably in the *pht4-7* mutants relative to the WT (Fig. 4B). Since upon chloroplastic Asc-deficiency increased NPQ was observed due to the increase of qI (Vidal-Meireles et al. 2020), these results suggest that Asc transport into the chloroplast was maintained in the *pht4-7* mutants.

We conducted state transition experiments using consecutive red and far-red illuminations (based on Ruban and Johnson 2009) in order to determine why NPQ was diminished in the *pht4-7* mutants. The *pht4-7* mutants displayed reduced qT, especially under high light conditions, although to a lesser degree than a *stt7* state transition mutant (Fleischmann et al. 1999; Fig. 4C; representative Chl *a* fluorescence traces can be found in Supplemental Fig. S6). This result raises the possibility that chloroplastic Pi may be decreased in the *pht4-7* mutants, since it has been described that state transition can be limited by Pi deficiency through insufficient LHCII phosphorylation (Petrou et al. 2008).

Next, measurements related to phosphate homeostasis were carried out. Phosphorous is taken up mostly in the form of Pi, therefore reduced Pi transport into the cell should decrease both the inorganic and organic phosphorous contents. We used Inductively Coupled Plasma Optical Emission spectroscopy (ICP-OES) to determine the total cellular phosphorous content and found that at normal light it was unaltered in the *pht4-7* mutants, whereas at high light, it was slightly diminished in the *pht4-7#7* mutant and augmented in the *pht4-7#9* mutant relative to the WT (Fig. 4D). Consequently, these data indicate that the absence of PHT4-7 did not limit phosphorous uptake into cells. On the other hand, inorganic phosphate is essential for ATP synthesis, and if CrPHT4-7 is a Pi transporter in the chloroplast envelope membrane, then its absence could limit ATP synthesis. Indeed, we found that cellular ATP content was lower in both *pht4-7* mutants, both in normal and high light conditions (Fig. 4E).

ATP production in the chloroplast is driven by transthylakoid proton motive force (pmf) that is physiologically stored as a ΔpH and a membrane potential (ΔΨ) (Cruz et al. 2005). Decreased chloroplastic phosphate availability, thereby ATP production (Carstensen et al. 2018) is expected to lead to increased pmf across the thylakoid membrane, especially in strong light (Cruz et al. 2005). As shown in Fig. 4F, total pmf is increased in both mutants at high light conditions, supporting this scenario.

**Figure 5.**
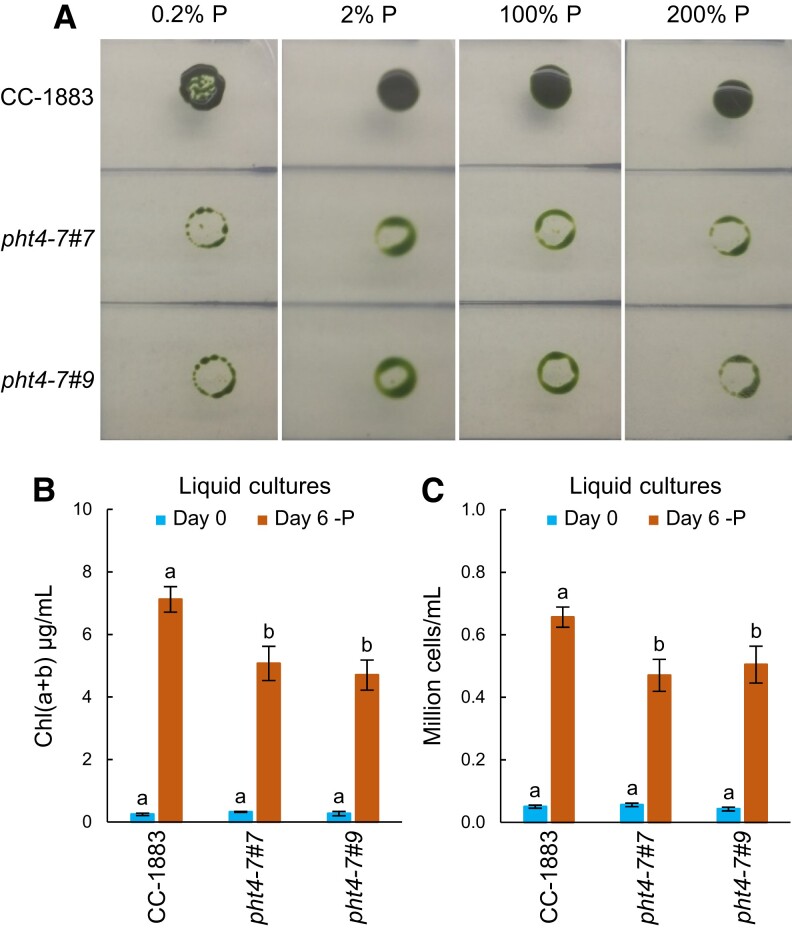
The effects of phosphorous deprivation on the wild type and the *pht4-7* mutants. **A)** Growth test of *pht4-7* mutants and the wild-type strain on TAP agar plates containing different amounts of phosphorous; the photos were taken after 6 d. **B)** Chl(a + b) contents at the beginning (Day 0) and after 6 d phosphorous deprivation (Day 6—P). **C)** Cell numbers at the beginning and after 6 d phosphorous deprivation. In panels **B)** and **C)**, liquid cultures were grown in Erlenmeyer flasks at 80 *µ*mol photons m^−2^ s^−1^. The averages and standard errors are based on 5 to 10 independent experiments with 1 to 2 biological replicates in each. The significance of differences between means was determined by ANOVA with Tukey post hoc test. The means with different letters are significantly different (*P* < 0.05).

In the subsequent step, we evaluated the response to varying Pi concentrations using spot tests. The growth of the *pht4-7* mutant strains was substantially hindered compared to the WT across low, normal, and high Pi levels (0.2, 2, 100, and 200% Pi of standard TAP medium, shown in Fig. 5A). These findings indicate that the lack of CrPHT4-7 leads to a consistent growth defect at all Pi concentrations, and this cannot be offset by doubling the Pi concentration in the media.

In liquid TAP cultures with 0.5% Pi, we observed a reduction in cell proliferation by approximately 30% in the *pht4-7* mutants compared to the WT, as assessed by the Chl(a + b) content and cell number of the cultures (Fig. 5, B and C). We note however that growth defect was in the same range under Pi replete conditions at normal light (Fig. 2), thus Pi limitation did not notably exacerbate the growth phenotype.

**Figure 6.**
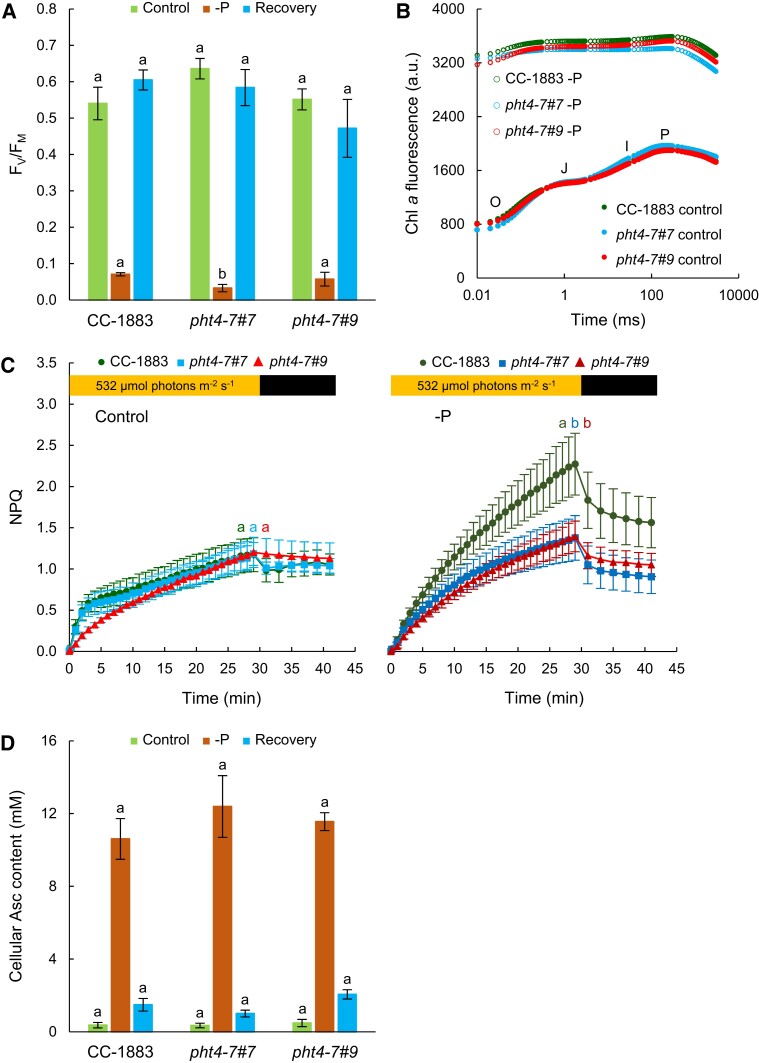
Alterations in photosynthetic activity upon phosphorous limitation. **A)***F_V_*/*F_M_* values of cultures grown in TAP for 6 d, and in TAP medium containing 0.5% P of regular TAP. For recovery, cultures were transferred to regular TAP media for 1 d. **B)** Fast Chl *a* fluorescence (OJIP) transients. **C)** NPQ (induced at 532 *µ*mol photons m^−2^ s^−1^) of cultures grown in regular TAP medium, and in 0.5% P containing TAP medium for 6 d. **D)** Total cellular Asc contents. All the cultures were grown in Erlenmeyer flasks at 80 *µ*mol photons m^−2^ s^−1^. The same Chl(a + b) amounts were set for the Chl *a* fluorescence measurements. The averages and standard errors are based on 3 to 5 independent experiments with 1 to 2 biological replicates in each. The significance of differences between means was determined by ANOVA with Tukey post hoc test. The means with different letters are significantly different (*P* < 0.05). In the case of panel **C)**, significance was calculated at the end of the illumination period.

Regarding the photosynthetic activity, we observed that after 6 d of Pi deprivation, there was a significant decrease in the *F_V_*/*F_M_* values across all genotypes, dropping to approximately 0.1 (Fig. 6A). Importantly, the decrease in *F_V_*/*F_M_* was caused by a very strong increase of the *F*_0_ value (Fig. 6B), indicating that the photosynthetic electron transport became reduced under Pi deprivation, possibly due to ATP deficiency (Fig. 4E), therefore a limited Calvin-Benson cycle activity. Upon the re-addition of Pi, the *F_V_*/*F_M_* was almost fully restored within 24 h, showing that the downregulation of photosynthetic activity was reversible (Fig. 6A). Moreover, upon Pi limitation, NPQ increased, with the increase being less substantial in the *pht4-7* mutants than in the WT (Fig. 6, C and D).

In addition, we have detected a very strong (about 20 to 30-fold) increase in Asc contents upon Pi limitation in each strain, which was substantially restored by the re-addition of Pi within 24 h (Fig. 6D). These data show that Pi limitation leads to a strong Asc accumulation, similarly to sulfur deprivation involving oxidative stress (Nagy et al. 2018), and that upon the release of this stress effect, the Asc content rapidly returns to its original level.

### Genetic complementation and overexpression of CrPHT4-7

**Figure 7.**
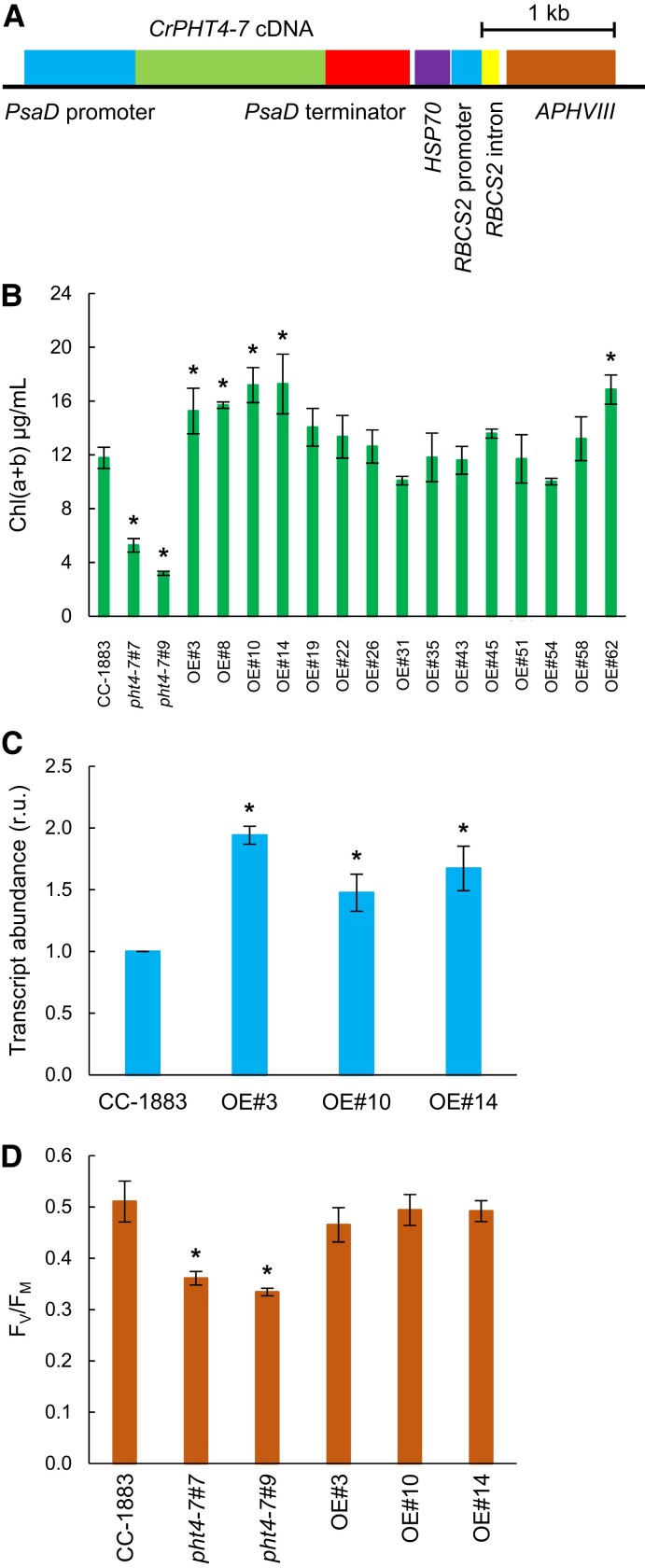
Overexpressing CrPHT4-7 in CC-1883 leads to improved growth in high light. **A)** Map of the pJR101 plasmid containing the coding sequence of *CrPHT4-7*, the strong *PSAD* promoter, the *APHVIII* resistance gene, and the *PSAD* terminator. **B)** Chl(a + b) contents of CC-1883, *pht4-7* mutants, and several randomly selected *pht4-7*-overexpressing lines after 3 d of growth at 500 *µ*mol photons m^−2^ s^−1^ in TAP medium in Erlenmeyer flasks. **C)***PHT4-7* transcript abundance in CC-1883 and the selected *pht4-7*-overexpressing lines (OE#3, OE#10, OE#14) **D)**, *F_V_*/*F_M_* values measured on the same cultures. The averages and standard errors are based on 3 to 6 independent experiments with 2 to 6 replicates in each. The significance of differences between means was determined by ANOVA with Dunnett’s post hoc test. Asterisks indicate significantly different means (*P <* 0.05) compared to the control strain CC-1883.

To confirm the relationship between the observed effects and the CrPHT4-7 mutation, genetic complementation experiments were carried out. We cloned the full-length *CrPHT4-7* cDNA between the promoter and terminator sequence of *PSAD*, and subsequently transformed the *pht4-7* mutants with this construct (Fig. 7A). The complementation rescued the slow growth phenotype of the *pht4-7* mutants in at least 70% of the transformants tested (randomly selected lines for the complemented *pht4-7#7* mutant are shown in Supplemental Fig. S7A). The restored growth phenotype was also associated with higher Chl(a + b)/mL contents, improved photosynthetic performance (as assessed by the *F_V_*/*F_M_* value), and moderate increases in Asc content, when grown in high light (Supplemental Fig. S7, B to D). Importantly, the complemented lines grew similarly upon Pi limitation in normal light as the WT (Supplemental Fig. S7E).

We also transformed CC-1883 with the above-mentioned construct to obtain *CrPHT4-7*-overexpressing lines. Out of 15 randomly selected lines, 5 showed significantly improved growth relative to the WT, as evidenced by higher Chl(a + b)/mL contents when grown in high light (Fig. 7B). The relative transcript abundance of *PHT4-7* was significantly increased in the selected overexpressing lines (Fig. 7C). The *F_V_*/*F_M_* values of the WT and the overexpressing lines did not differ significantly under high light treatment (Fig. 7D), indicating that the performance of the photosynthetic apparatus was similar in the overexpressing lines and the WT.

### Expression of CrPHT4-7 in a yeast strain lacking phosphate transporters

In order to study the substrate specificity of CrPHT4-7, we used the EY917 yeast strain in which 5 Pi transporters (PHO84, PHO87, PHO89, PHO90, PHO91) were inactivated, and the GAL1 promoter drives the expression of *PHO84* enabling growth on galactose-containing media (Wykoff and O'Shea 2001). The EY917 strain lacking the 5 Pi transporters is considered conditional lethal, because spores are unable to germinate in the absence of galactose (i.e. on normal glucose-containing growth media). Plant phosphate transporters have been successfully investigated using Pi transporter-deficient yeast strains (Wang et al. 2015, Chang et al. 2019).

**Figure 8.**
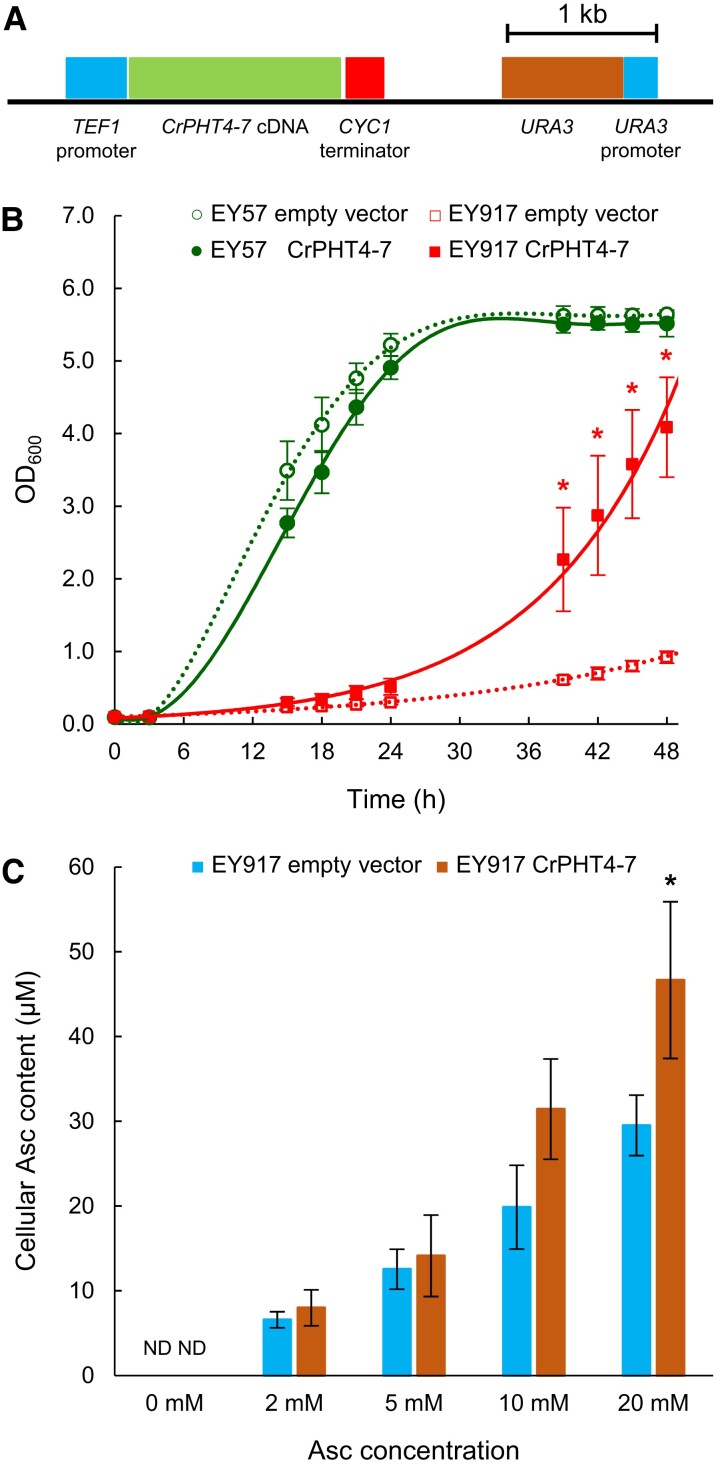
CrPHT4-7 transports phosphate in a yeast experimental system. **A)** Physical map of the construct for heterologous complementation. **B)** Growth rates of strain EY57 and the phosphate-transporter-deficient strain EY917 expressing the empty vector or CrPHT4-7. **C)** Uptake of ascorbate (Asc) into yeast cells expressing CrPHT4-7 in comparison to the control strain. The cultures were incubated with 0, 2, 5, 10, and 20 mm Asc for 15 min. The averages and standard errors are based on 3 to 4 independent experiments. Data were analyzed by Welch's unpaired *t*-test. Asterisks indicate significantly different means (*P <* 0.05) compared to the respective empty vector-containing strain. ND, non-detectable.

We transformed the EY57 (WT) and EY917 strains with the p426-TEF plasmid containing the *CrPHT4-7* gene (Fig. 8A). As controls, we used the EY57 and EY917 yeast strains transformed with the empty vector. The effect of expressing *CrPHT4-7* on the growth characteristics was then analyzed on glucose-containing medium. We found that the growth of the yeast strain expressing CrPHT4-7 was remarkably improved relative to the EY917 empty vector strain. Expressing CrPHT4-7 in the control strain EY57 had no significant effect on its growth properties in comparison with the EY57 empty vector strain (Fig. 8B). These data demonstrate that CrPHT4-7 acts as a Pi transporter.

Since CrPHT4-7 is also a potential Asc transporter because it shares high similarity with the Asc transporter AtPHT4;4, its Asc uptake activity was also investigated in yeast cells. To this end, yeast cultures expressing CrPHT4-7 or an empty vector were incubated in the presence of 2, 5, 10, and 20 mm Na-Asc for 15 min. The control cultures contained no Asc (Fig. 8C), in agreement with published reports that yeast contains no Asc, but erythroascorbate instead (Spickett et al. 2000). At the 2 lowest concentration levels (2 and 5 mm), no significant difference between the EY917 and the CrPHT4-7 expressing yeast strains were observed (Fig. 8C). At 10 and 20 mm concentration levels, the uptake in the CrPHT4-7 expressing strain was more enhanced; however, the intracellular Asc concentration was only approx. 20 to 50 *µ*m, i.e. 0.2% of the external Asc level (Fig. 8C). This shows that Asc uptake by yeast cells is very moderate and it is only slightly increased by CrPHT4-7. We also note that the regular Asc content in *C. reinhardtii* in the range of 0.1 to 1 mm (Vidal-Meireles et al. 2017, and Fig. 3A), making it also unlikely that CrPHT4-7 substantially contributes to Asc content into the chloroplasts of *C. reinhardtii*. These data are in agreement with our results obtained with Chlamydomonas cells (Fig. 3B) and indicate that CrPHT4-7 does not act as an effective Asc transporter.

## Discussion

Transporters play an essential role in Pi uptake and distribution within the cell. The Pi transporters situated in the cytoplasmic membrane of the cell are divided into 2 categories according to their affinity to the translocated Pi: There are low-rate high-affinity and high-rate low-affinity transporters, of which high-affinity Pi transporters are upregulated during P shortage (Grossman and Aksoy 2015). These include putative H^+^/PO_4_^3−^ PTA and Na^+^/PO_4_^3−^ PTB symporters (Moseley et al. 2006, Wang et al. 2020, Sanz-Luque and Grossmann 2023). In addition to PTA and PTB transporters, PHT3 and PHT4 transporters have also been identified by genetic analysis (e.g. Wang et al. 2020), but to our knowledge, none have been characterized in detail.

We found that CrPHT4-7, a member of the PHT4 family in *C. reinhardtii*, is a Pi transporter localized to the chloroplast envelope membrane (Fig. 1). The *pht4-7* mutants exhibited slow growth under mixotrophic conditions, particularly in high light (Fig. 2). However, under photoautotrophic conditions that restrict growth, we observed no noticeable differences between the WT and the *pht4-7* mutants in terms of growth rate, *F_V_*/*F_M_*, and NPQ (Supplemental Fig. S4). Additionally, we found that the total phosphorous content was not affected by the absence of CrPHT4-7 (Fig. 4D), and providing the cultures with extra Pi did not mitigate the growth defect (Fig. 5). Interestingly, phosphorous limitation did not notably enhance the phenotype of the *pht4-7* mutants compared to the WT (Fig. 5). These observations suggest that only chloroplastic Pi uptake was affected by the absence of CrPHT4-7, not cellular Pi uptake. Under conditions conducive to high growth rate (i.e. in TAP medium), the absence of CrPHT4-7 resulted in a relative chloroplastic Pi deficiency and a shortage of ATP (Fig. 4E), thereby limiting Calvin-Benson cycle activity and leading to slower growth. Concurrently, the photosynthetic electron transport chain became over-reduced, as indicated by the increased pmf in high light (Fig. 4F). The decrease of the *F_V_*/*F_M_* value in high light conditions (Fig. 2H) suggests that in addition to the over-reduced electron transport chain, PSII may also become photoinhibited. These findings illustrate that CrPHT4-7 plays a crucial role in maintaining an adequate level of Pi in the chloroplast, thereby enhancing cellular fitness.

In relation to the impact of the absence of CrPHT-7 on photosynthesis, comparable findings have been reported during Pi deficiency in green algae and vascular plants (Wykoff et al. 1998, Petrou et al. 2008; Carstensen et al. 2018), as well as in a *pht2;1* mutant of wheat (*Triticum aestivum*) (Guo et al. 2013). Ribosome degradation and a decrease in photosynthetic electron transport activity, including the loss of the PSII subunit, PsbA, has been also observed upon Pi deficiency (Wykoff et al. 1998). The downregulation of photosynthetic electron transport serves to minimize photodamage, as the diminished activity of the Calvin-Benson cycle means that much of the absorbed light energy cannot be utilized to support cellular metabolism. Under conditions of P starvation, several photoprotective mechanisms, as well as P storage and uptake mechanisms, are activated (reviewed by Sanz-Luque and Grossman 2023).

Phosphate transporter mutants of vascular plants display enhanced NPQ due to a higher ΔpH induced by ATP limitation (Guo et al. 2013; Karlsson et al. 2015). By contrast, in our *pht4-7* mutants, NPQ decreased when grown in high light. NPQ mechanisms in green algae differ in many respects from those in vascular plants (Erickson et al. 2015, Vecchi et al. 2020). qE, which is a rapid ΔpH-dependent component appearing mostly under photoautotrophic growth conditions (Erickson et al. 2015), was not induced under our conditions (Fig. 4). Instead, NPQ developed on a timescale of several minutes, which may include the zeaxanthin-dependent (qZ), state transition-related (qT), and photoinhibitory (qI) components of NPQ (Erickson et al. 2015, Vidal-Meireles et al. 2020). We observed that pmf was elevated in both *pht4-7* mutants, suggesting that the decreased NPQ was not due to lack of membrane energization. On the other hand, ATP production and state transition (responsible for the qT component) were diminished in the *pht4-7* mutants (Fig. 4), most probably due to a limited Pi availability (as observed previously in *Dunaliella* upon Pi starvation, Petrou et al. 2008). Compromised state transition, acting as a major photoprotective mechanism in green algae (e.g. Goldschmidt-Clermont and Bassi 2015), may also explain the diminished *F_V_*/*F_M_* values in the *pht4-7* mutants grown at high light (Fig. 2).

The apparent Pi limitation in the chloroplast led to a dramatic increase in cellular Asc content when the cultures were grown in high light (Fig. 3A). The high-level accumulation of Asc in the *pht4-7* mutants may occur to mitigate reactive oxygen species, as provoked by compromised state transition and ATP synthesis diminishing CO_2_ fixation. When accumulating to high levels, Asc may also inactivate the OEC to alleviate the consequences of over-reduction of the electron transport chain when CO_2_ assimilation is impaired (Nagy et al. 2018). Thus, it seems that chloroplastic Pi deficiency triggers high Asc accumulation in *C. reinhardtii*, similar to induction of Asc accumulation upon sulfur deprivation (Nagy et al. 2016). Conversely, overexpression of *CrPHT4-7* in *C. reinhardtii* resulted in enhanced resistance to high light stress, demonstrating that Pi transport can limit photosynthesis under intensive illumination.

Although CrPHT4-7 exhibits a relatively high degree of similarity with AtPHT4;4, it did not show substantial Asc transport activity. In algal cells, Asc uptake into the chloroplasts, as tested by incubating the cultures with Asc and measuring Chl *a* fluorescence transients, did not seem to differ between the WT and the *pht4-7* mutants. When expressed in yeast, CrPHT4-7 did not enhance Asc uptake into the cells in the physiologically relevant concentration range (Figs. 3 and 8). At high concentrations, there was a slight enhancement of Asc uptake by the CrPHT4-7 transporter; however, physiologically, it is probably of little importance.

In summary, we have shown that CrPHT4-7 supports Pi homeostasis and photosynthesis in the chloroplasts and overexpressing CrPHT4-7 enhanced high light tolerance. On the other hand, the loss of CrPHT4-7 function was not lethal even though Pi is essential to maintain chloroplast function. It thus appears likely that there are additional PHT transporters located in the chloroplast envelope membrane. PHT2 transporters are not found in green algae (Bonnot et al. 2017), therefore, other members of the PHT4 family are likely to supply Pi to chloroplasts, as suggested also by in silico analysis (Wang et al. 2020, 2023). Confirming the identity and revealing the physiological roles of additional chloroplastic Pi transporters should be the subject of future studies. Pi transporters located in the plasma membrane, the mitochondria, and other cellular compartments have not been characterized in detail in green algae either; their analysis will be important to fully exploit the so-called “luxury uptake” characteristics of green algae toward mitigating excess Pi in polluted waters and for the development of wastewater treatment strategies.

## Materials and methods

### Algal strains

The *pht4-7#7* and *pht4-7#9* mutant strains of *C. reinhardtii* were generated via CRISPR/Cas12a, published previously, using CC-1883 as the background strain (Ferenczi et al. 2017). To generate complementation and *PHT4-7* overexpressing lines, the coding sequence of the *CrPHT4-7* gene was synthesized (GeneCust, Boynes, France) with NdeI and EcoRI restriction sites at the 5′ and 3′ ends, respectively. The fragment was cloned into the similarly digested vector pJR39 (Neupert et al. 2009), generating the transformation vector pJR101. Nuclear transformation of the CC-1883 and *pht4-7#7* strains of *C. reinhardtii* was performed using the glass bead method (Neupert et al. 2012). Selection was performed on TAP plates supplemented with 10 *µ*g/mL paromomycin.

### Generation of PHT4-7 expressing yeast strains

We used the EY57 (*MATa ade2-1 trp1-1 can1-100 leu2-3,112 his3-11,15 ura3*) and the EY917 (*MATα ade2-1 trp1-1 can1-100 leu2-3,112 his3-11,15 ura3 pho84::HIS3 pho87::CgHIS3 pho89::CgHIS3 pho90::CgHIS3 pho91::ADE2, pGAL1-PHO84* (EB1280)) *Saccharomyces cerevisiae* strains that were kindly provided by Dr. Dennis Wykoff (Villanova University, USA).

The coding sequence of the *CrPHT4-7* gene with BamHI and EcoRI restriction sites at the 5′ and 3′ ends was cloned into the similarly digested vector p426-TEF (containing *URA3* marker), generating the transformation plasmid. We transformed EY57 and EY917 *S. cerevisiae* strains with the plasmid containing the *CrPHT4-7* gene by selecting for the *URA3* marker. We followed the transformation protocol by Gietz and Schiestl (2007). For transformation, strains were grown in synthetic media lacking uracil and containing 2% (w/v) galactose.

### Structure prediction of PHT4-7 and sequence alignment

To predict the transmembrane helices of CrPHT4-7, we used the TMHMM v. 2.0 (Krogh et al. 2001), Deep TMHMM v. 1.0.24 (Hallgren at al. 2022), and the Phyre^2^ v. 2.0 (Kelley et al. 2015) online software. Amino acid sequence alignment was performed by MultAlin (Corpet 1988).

### Growth of alga cultures

Precultures were grown mixotrophically in Tris-acetate-phosphate medium (TAP, Gorman and Levine 1965) in 25 mL Erlenmeyer flasks for 3 d on a rotatory shaker at 130 rpm, at 23 °C and 80 *µ*mol photons m^−2^ s^−1^, measured at the top of the flasks. By the third day of growth in TAP, a cell density of 2 to 4 million cells/mL was reached.

For the assessment of culture growth parameters (in Fig. 2), the precultures were diluted to 0.5 *µ*g Chl(a + b)/mL and were placed in a Multi-Cultivator MC 1000-OD instrument (Photon Systems Instruments, Brno, Czech Republic). The cultures were grown for up to 3 d at 23 °C with intense air bubbling, at a light intensity of 60 or 350 *µ*mol photons m^−2^ s^−1^ measured within the culture tubes.

For measuring the rest of the physiological measurements (e.g. photosynthetic parameters, ATP, and Asc contents), the cultures were grown in 50 mL Erlenmeyer flask for 3 d on a rotatory shaker at 130 rpm, 23 °C. For most experiments, the cultures were grown in TAP medium, and in a subset of experiments, HS medium was used. The initial Chl concentration was 0.5 *µ*g Chl(a + b)/mL, and the light intensity was 80 or 500 *µ*mol photons m^−2^ s^−1^, measured at the top of the flasks (the effective light intensity is remarkably lower within the flask). We noted that shake-flask culturing was less stressful for the cells than growth in the Multi-Cultivator MC 1000-OD instrument.

### Growth of yeast cultures for CrPHT4-7 expression

In order to enable the growth of the EY917 strain (containing *GAL1-PHO84*), precultures for both strains (EY57 and EY917) were grown in synthetic yeast media with 2% (w/v) galactose and appropriate amino acids for 1 d on a rotatory shaker at 30 °C. To prevent *PHO84* expression, the precultures were harvested by centrifugation (3,000 × *g*, 1 min, 25 °C), washed 2 times, and were diluted to OD_600_ = 0.1 with synthetic yeast media containing 2% (w/v) glucose and appropriate amino acids without uracil. The cultures were grown for 2 d on a rotatory shaker at 140 rpm at 30 °C.

### Chlorophyll and Asc content measurements and phosphorus content determination in *C. reinhardtii*

Chl(a + b) content was determined according to Porra et al. (1989), and the Asc content was determined as in Kovács et al. (2016). Total phosphorus content determination was performed by ICP-OES, as described in Nagy et al. (2018).

### ATP content determination

ATP was measured using the Adenosine 5′-triphosphate (ATP) Bioluminescent Assay Kit (Sigma-Aldrich) according to the instructions of the manufacturer. 3 × 10^7^ algal cells were harvested by centrifugation (21,130 × *g*, 1 min, 4 °C) and washed once with ice cold sterile water. The pellets were resuspended in 250 *µ*L ice cold sterile water. Cells were broken by vortexing for 2 min with 80 *µ*L quartz sand. After the vortexing, the samples were centrifuged (21,130 × *g*, 1 min, 4 °C). 200 *µ*L of the supernatant were transferred into EZ-10 Spin Columns (Bio Basic Inc.) and rapidly spun down (21,130 × *g*, 1 min, 4 °C). Until ATP determination, the samples were stored on ice. The cellular ATP concentration was determined using a cell volume of 140 fL (Craigie and Cavalier-Smith 1982).

### Phosphorus deprivation

Precultures were grown mixotrophically in TAP medium in 50 mL Erlenmeyer flasks for 3 d on a rotatory shaker at 130 rpm, 23 °C and 80 *µ*mol photons m^−2^ s^−1^. After 3 d, the cells were harvested by centrifugation (3,000 × *g*, 1 min, 23 °C), washed 3 times, and were diluted to 0.5 *µ*g/mL Chl(a + b) with 0.5% (5.1 *µ*m) Pi-containing TAP medium. The cultures were grown at 23 °C, 80 *µ*mol photons m^−2^ s^−1^, on a rotatory shaker at 130 rpm, for 6 d.

### Drop test

The growth characteristics of the strains were tested on TAP agar plates, containing different amounts of phosphorus (2.04 *µ*m—0.2%; 20.4 *µ*m—2%; 1.02 mm—100% 2.04 mm—200%). Precultures were grown mixotrophically in TAP medium in 50 mL Erlenmeyer flasks for 3 d on a rotatory shaker at 130 rpm, 23 °C and 80 *µ*mol photons m^−2^ s^−1^. After 3 d, the cells were harvested by centrifugation (3,000 × *g*, 1 min, 23 °C), washed 3 times, and were diluted to 5 *µ*g/mL Chl(a + b) with 0.5% (5.1 *µ*m) Pi-containing TAP medium. Ten microlitres of each algal strain was dropped onto the agar plates. The plates were incubated at 23 °C for 6 d. The intensity of illumination was 80 *µ*mol photons m^−2^ s^−1^.

### Ascorbate uptake measurements in *C. reinhardtii* and yeast

The 3-d-old *C. reinhardtii* precultures (in TAP medium) were diluted to 10 *µ*g/mL Chl(a + b), and incubated for 2 h on a rotatory shaker at 23 °C and 80 *µ*mol photons m^−2^ s^−1^ with or without 20 mm Asc.

Yeast cultures were kept in yeast synthetic media with 2% glucose (w/v) and appropriate amino acids for 1 d on a rotatory shaker at 30 °C. After 1 d, we measured the OD_600_ values of the cultures (the strains were grown to log phase OD_600_ = 0.7 to 1.5), and set OD_600_ = 0.8. We added 0, 2, 5, 10, and 20 mm Asc, and incubated the cultures for 15 min on a rotatory shaker at 30 °C. We harvested the cells by centrifugation (3,000 × *g*, 1 min, 4 °C), washed 3 times with 40 mL ice cold synthetic media, and immediately frozen in liquid nitrogen. Cells were broken by vortexing for 30 s with glass beads (425 to 600 *µ*m, Sigma-Aldrich, St. Louis, USA). The Asc content was determined as in Kovács et al. (2016) with slight modifications.

### Analysis of gene expression

For isolation of RNA, 2 mL of cultures was harvested and Direct-Zol RNA MiniPrep kit (Zymo Research) was used, following the recommendations of the manufacturer. To remove contaminating DNA from the samples, the isolated RNA was treated with DNaseI (Zymo Research). RNA integrity was checked on a 1% (w/v) denaturing agarose gel. One microgram of total RNA was used for cDNA synthesis with random hexamers using FIRESript reverse transcriptase (Solis BioDyne). To confirm the absence of DNA contaminations, an aliquot of the RNA sample was used without reverse transcriptase. Reverse transcription quantitative PCR (RT-qPCR) analysis was performed using a Bio-Rad CFX384 Touch Real-Time PCR Detection System, using HOT FIREPol EvaGreen qPCR Mix Plus (Solis Biodyne) for cDNA detection. The primer pairs for the reference genes (*actin* [Cre13.g603700], *β-Tub2* [Cre12.g549550], *CBLP* [Cre06.g278222], *UBQ2* [Cre09.g396400]) used in RT-qPCR were published earlier in Vidal-Meireles et al. (2017). For *PHT4-7*, 5′-CAACTGGGGCTACTACACGC-3′ forward and 5′-CCATGACCCGCTCCTCATATC-3′ reverse primers were used. The data are presented as fold-change in mRNA transcript abundance, normalized to the average of the reference genes, and relative to the WT sample. RT-qPCR analysis was carried out with 3 technical replicates for each sample and 3 to 4 biological replicates, originating from independent experiments were analyzed. The standard errors (Se) were calculated based on the different transcript abundances amongst the independent biological replicates.

### Determination of cell size and cell number

The cell size and cell number were determined by a Luna-FL dual fluorescence cell counter (Logos Biosystems Inc.).

### Chl *a* fluorescence measurements

Fast Chl *a* fluorescence measurements were carried out with a Handy-PEA instrument (Hansatech Instruments Ltd, King's Lynn, UK), as described in Nagy et al. (2018).

NPQ was measured using a Dual-PAM-100 instrument (Heinz Walz GmbH). *C. reinhardtii* cultures were dark adapted for 30 min on a rotatory shaker; then, liquid culture containing 40 *µ*g Chl(*a* + *b*)/mL was filtered onto Whatman glass microfiber filters (GF/B) that were placed between 2 microscopy coverslips with a spacer to allow for gas exchange. For NPQ induction, light adaptation consisted of 30 min illumination at 532 *µ*mol photons m^−2^ s^−1^, followed by 12 min of dark adaptation interrupted with saturating pulses of 3,000 *µ*mol photons m^−2^ s^−1^.

For analyzing state transition, actinic red light (AL, 15 *μ*mol photons m^−2^ s^−1^) and far red (FR) light (255 *μ*mol photons m^−2^ s^−1^) were employed for 15 min (phase 1) on dark-adapted cultures. After this phase, the far red light was turned off and only red light illumination was employed for 15 min to induce state II (phase 2). Finally, we used again the red light—far red light combination for 15 min to drive the state II—state I transition (phase 3). During the measurement, saturating light pulses (8,000 *μ*mol photons m^−2^ s^−1^ for 600 ms) were given every minute. qT parameter was calculated as: qT = (*F_M_*^I^—*F_M_*^II^)/*F_M_*^II^, in which *F_M_*^I^ was determined at the end of the phase 3, and *F_M_*^II^ at the end of the phase 2.

### Pmf measurements

Estimation of the transthylakoid pmf was carried out by Dual-PAM-100 system with the P515/535 extended emitter-detector modules (Schreiber and Klughammer 2008). Before the measurement, samples were kept for 10 min in darkness, and cultures equivalent to 40 *µ*g/mL Chl(a + b) were filtered onto a GF/C filter paper. Samples were placed between 2 object slides with a spacer to allow for gas exchange. Samples were illuminated with 190 *µ*mol photons m^−2^ s^−1^ actinic red light for 2 min, then actinic light was switched off. The absorbance change at 515 nm against the 535 nm reference wavelength was recorded during the light-dark transition (Cruz et al. 2001; Kramer and Sacksteder 1998). The change of signal was expressed in ΔI/I units (Schreiber and Klughammer 2008).

### Generation of PHT4-7-Venus expressing lines and localization of PHT4-7 in Chlamydomonas

Nuclear transformation of strains UVM11 and CC-4533 (also known as cMJ030) of *C. reinhardtii* with the plasmid pLM005-CrPHT4-7 was done using the glass bead method (Neupert et al. 2012). The base plasmid pLM005 was previously used in the study by Wang et al. (2023). We also transformed the CC-1883 strain and the *pht4-7* mutants with this construct, but failed to obtain transgenic clones showing a clear Venus signal, most probably due to very low expression levels caused by epigenetic transgene silencing (Neupert et al. 2020).

The pLM005-CrPHT4-7 plasmid contains the full-length CrPHT4-7 gene including the introns (length: 3458 bp). The plasmid was linearized using the restriction enzyme ScaI. Precultures of the transformed strains were grown mixotrophically in TAP medium in 25 mL Erlenmeyer flasks for 3 d. The strains were then transferred to Tris-phosphate (TP) medium and further grown for 16 h under the above-mentioned conditions, after which the cells were immobilized in 0.8% low-melt agarose (Carl Roth, Karlsruhe, Germany) before imaging. Imaging was performed using a Leica TCS SP8 confocal laser scanning microscope with a hybrid detector (Leica, Heidelberg, Germany). Single optical sections were taken using HCPLAPO CS2 63× (NA:1.2) water immersion objective with a working distance of 0.3 mm. Microscope configuration was as follows: scan speed: 200 Hz; line averaging: 4; scanning mode: unidirectional; pinhole: 111.4 *µ*m; zoom: 7×; and excitation: 514 nm laser for Venus-CrPHT4-7 at 12.7% laser intensity, 552 nm laser for Chl auto-fluorescence at 8.5% laser intensity. Venus-CrPHT4-7 fluorescence and Chl auto-fluorescence were detected between 520 and 540 nm and 650 and 750 nm, respectively. HyD SP GaAsP detector was used to detect the Venus-CrPHT4-7 signal. Images were pseudocolored and analyzed using Leica LAS AF software (version 2.6) and ImageJ (version 1.53k).

### Statistics

The presented data are based on at least 3 independent experiments. When applicable, averages and standard errors (±Se) were calculated. Statistical significance was determined using Welch's unpaired *t*-test (GraphPad Prism v. 10.0.2.232 online software), ANOVA with Tukey post hoc test (OriginPro 2020b software), or Dunnett's post hoc test (IBM SPSS Statistics v. 25.0 software). Changes were considered statistically significant at *P* < 0.05.

### Accession numbers

The accession numbers for *C. reinhardtii PHT4-7* (also called *PHT7*) and *STT7* are Cre16.g663600 and Cre02.g120250, respectively. The accession numbers for *A. thaliana PHT4;1*, *PHT4;2*, *PHT4;3*, *PHT4;4*, *PHT4;5*, and *PHT4;6* are At2g29650, At2g38060, At3g46980, At4g00370, At5g20380, and At5g44370, respectively.

## Supplementary Material

kiad607_Supplementary_Data

## Data Availability

All data presented in this study are available within this article or Supplemental Materials. There are no special databases associated with this manuscript.

## Supplemental data

The following materials are available in the online version of this article.

**Supplemental Figure S1**. Amino acid sequence alignment of members of the PHT4 family in Arabidopsis (AtPHT4) and CrPHT4-7 in *C. reinhardtii*.

**Supplemental Figure S2**. Subcellular localization of CrPHT4-7 in the CC-4533 strain.

**Supplemental Figure S3**. Culture growth of independent *pht4-7* mutant lines generated by the CRISPR/Cas12a technique in TAP medium in continuous illumination in a Multi-Cultivator photobioreactor.

**Supplemental Figure S4**. Phenotype of *pht4-7* mutants under photoautotrophic growth conditions.

**Supplemental Figure S5**. Cell number and chlorophyll values of *pht4-7* mutants and the wild type grown in Erlenmeyer flasks.

**Supplemental Figure S6**. Typical state transition kinetics of *pht4-7* and *stt7* mutants.

**Supplemental Figure S7**. Complementation of the *pht4-7#7* CRISPR/Cas12a mutant.

## Dive Curated Terms

The following phenotypic, genotypic, and functional terms are of significance to the work described in this paper:

PHT3 Gramene: Cre03.g172300
PHT3 Araport: Cre03.g172300

PHT4;2 Gramene: AT2G38060
PHT4;2 Araport: AT2G38060

PHT4;3 Gramene: AT3G46980
PHT4;3 Araport: AT3G46980

PHT4;5 Gramene: AT5G20380
PHT4;5 Araport: AT5G20380

PHT4;6 Gramene: AT5G44370
PHT4;6 Araport: AT5G44370

PHT2;1 Gramene: AT3G26570
PHT2;1 Araport: AT3G26570

ATP CHEBI: CHEBI:15422

PHT4;4 Gramene: At5g20380
PHT4;4 Araport: At5g20380

PHT4-7 Gramene: Cre16.g663600
PHT4-7 Araport: Cre16.g663600

STT7 Gramene: Cre02.g12025
STT7 Araport: Cre02.g12025
