# Supplementary material for: Chloroplast phosphate transporter CrPHT4-7 regulates phosphate homeostasis and photosynthesis in Chlamydomonas
Source: Plant Physiol. 2023 Nov 14;194(3):1646–61. doi: 10.1093/plphys/kiad607 (PMC10904345; doi:10.1093/plphys/kiad607)
Supplement: kiad607_Supplementary_Data [file kiad607_supplementary_data.pdf]

```
CrPHT4-7   ---------- ---------- ---------- ---------- ---------- ----------
AtPHT4;4   MALGGLISNR NFGSFIGSGN GCQRLGKSGA EVSKLFPNAL LCRNHQPLQA SLHHESGHMR   60
AtPHT4;5   ---------- ---------- ---MARLTLR PHNHFFSSPI YAHKQPFLSV YTIFPHHHQ-   36
AtPHT4;2   ---------- ------MATV GSLKPLHHSS CSSSFPRNPI V--NRKALLG FVF----DSA   38
AtPHT4;1   ---------- ---------- -----MNARA LLCSSNIHSL YTSNRPPEKT SSSRSLRNLK   35
AtPHT4;6   ---------- ---------- ---------- ---------- ---------- ----------
AtPHT4;3   ---------- ---MCYSLSI QSSIDFHNRN ALKIHGDRAI LTSNLPTLRR IPFLPERDRR   47

CrPHT4-7   ---------- ---------- ---------- ---------- ---------- ----------
AtPHT4;4   RSFGCFLQPR MDSVIRFRNS IKINRSRAYY KSEESDITEG VVPSADGSAE AILVEGNLQN   120
AtPHT4;5   ---------N PLIKSRVKCS ASGTERVRES KKLPPKDPIE DPKPQLPIPE VLSTETGFEQ   87
AtPHT4;2   RK-------N QIRCENLRYS SESDGKRR-N AAAKKRNQSP ERCAAEGVLT GGGGSEAIAE   90
AtPHT4;1   PSPKSL---R VWIYPRNRSS VFRVLVRSSD KSESSNSYYV EGDKVSGNND -VVSDSPSSI   91
AtPHT4;6   ---------- ---------- ---------- ---------- ---------- ----------
AtPHT4;3   RK-------L VLCTGRVVNS LKFTGNTSVD LCGIPRHRLR VSCSDARRTP EETAAELTAQ   100

CrPHT4-7   ----MNTRPH RWNVVVMIAV AFVLCNMDKV NMSVAVIPMA AELGWSATER GLVSSSFFWG   56
AtPHT4;4   ASPWWQQFPR RWVIVLLCFS SFLLCNMDRV NMSIAILPMS QEYNWSSATV GLIQSSFFWG   180
AtPHT4;5   NWPPWKNIPQ RYKLIGATSL AFVICNMDKV NLSIAIIPMS HQFGWSSSVA GLVQSSFFWG   147
AtPHT4;2   VRT---MMPE RIKVVILTAC MMCLCNADRV VMSVAVVPLA DKLGWSSSFL GVVQSSFLWG   147
AtPHT4;1   VLPWWEEFPK RWVIVLLCFS AFLLCNMDRV NMSIAILPMS AEYGWNPATV GLIQSSFFWG   151
AtPHT4;6   --MKLSNIPQ RYVIVFLTFL STCVCYIERV GFSIAYTVAA DAAGINQSSK GTILSTFFVG   58
AtPHT4;3   PNFSEFITSE RVKVVAMLAL ALALCNADRV VMSVAIVPLS LSRGWSKSFS GIVQSSFLWG   160

CrPHT4-7   YSATQLPAGY ISTKIGGAKV LAAGVALWSF GTLIAPPAAQ TSLLALCATR VLVGLGEGFA   116
AtPHT4;4   YLLTQILGGI WADKFGGKVV LGFGVVWWSF ATIMTPIAAR LGLPFLLVVR AFMGIGEGVA   240
AtPHT4;5   YALSQLPGGW LSKIFGGRKV LEIGVFTWSF ATALVPLLAG F-MPGLIFSR ILVGIGEGVS   207
AtPHT4;2   YIFSSVIGGA LVDRYGGKRV LAWGVALWSL ATLLTPWAAA HSTLALLCVR AFFGLAEGVA   207
AtPHT4;1   YLLTQIAGGI WADTVGGKRV LGFGVIWWSI ATILTPVAAK LGLPYLLVVR AFMGVGEGVA   211
AtPHT4;6   YACSQVPGGW AAQKIGGRKV LLLSFVLWSS TCFLVPLDPN R-VGLLVVAR LLVGVAQGFI   117
AtPHT4;3   YLISPIAGGT LVDRYGGKVV MAWGVALWSL ATFLTPWAAD SSLWALLAAR AMVGVAEGVA   220

CrPHT4-7   PSAATAVLAK LVPSTERSRA VAAVWGGLDV GSAVGLLLCG PLIRMFGWPS VFYLFAVLGL   176
AtPHT4;4   MPAMNNMLSK WIPVSERSRS LALVYSGMYL GSVTGLAFSP MLITKFGWPS VFYSFGSLGS   300
AtPHT4;5   PSAATDLIAR TIPVKERSRA VGFVFGGLSL GSVMGLLLAP PIIETFNWES VFYLFGLLGV   267
AtPHT4;2   MPSMTTLLSR WFPMDERASA VGISMAGFHM GNVVGLLLTP LMLSSIGISG PFILFASLGL   267
AtPHT4;1   MPAMNNILSK WVPVQERSRS LALVYSGMYL GSVTGLAFSP FLIHQFGWPS VFYSFGSLGT   271
AtPHT4;6   FPSIHTVLAQ WVPPHERSRL VSITTSGMYL GAALGMWLLP ALVELRGPES VFLAEALAGV   177
AtPHT4;3   LPCMNNMVAR WFPPTERSRA VGIAMAGFQL GNVVGLMLSP ILMSQGGIYG PFVIFGLSGF   280

CrPHT4-7   VWVAAWPLVQ PDKMDPDMIA ENKKKEQERA TRLALAQANE VAASALESVD ETPVSATYAK   236
AtPHT4;4   IWFLLWLKFA YSSPKDDPDL SEEEKKVILG GS-------- ---------- ----------   332
AtPHT4;5   GWFVGFQFLN EEEVSYKGNE ISTSHKSENA TKE------- ---------- ----------   300
AtPHT4;2   LWVSTWSSGV TNNPQDSPFI TRSELRLIQA GKPV---QP- ---------- ----------   303
AtPHT4;1   VWLTLWLTKA ESSPLEDPTL LPEERKLIAD NC-------- ---------- ----------   303
AtPHT4;6   IWSLLWIRYA TDPPRSEHPK AAAAGFGGAL LPTN------ ---------- ----------   211
AtPHT4;3   LWLLVWLSAT SSAPDRHPQI TKSELEYIKQ KKQISTMEN- ---------- ----------   319

CrPHT4-7   LEKSLPPDGK VPWGEFFRSP PVWAVTVAHF CFNWGYYTLL AWLPSYFELA LGLNVERSSF   296
AtPHT4;4   --KPREPVTV IPWKLILSKP PVWALIISHF CHNWGTFILL TWMPTYYNQV LKFNLTESGL   390
AtPHT4;5   --ELGSSLKE IPWKSFFQSP AVWAMIYTHF CGSWGHYTCL SWLPTYFSEA LSLNLTEAAW   358
AtPHT4;2   --STISPKPN PSLRLLLSKL PTWAIIFANV TNNWGYFVLL SWMPVYFQTV FNVNLKQAAW   361
AtPHT4;1   --ASKEPVKS IPWRLILSKP PVWALISCHF CHNWGTFILL TWMPTYYHQV LKFNLMESGL   361
AtPHT4;6   --VNHHKVTH IPWKKIMLSL PVWAIVVNNF TFHYALYVLM NWLPTYFELG LQISLQGMDS   269
AtPHT4;3   --KRISTSGI PPFGRLLSKM PTWAVIVANS MHSWGFFVIL SWMPIYFNSV YHVNLKQAAW   377

CrPHT4-7   LTLIPYIAMT AMMPLVGPVA DGWVKNGV-P LTRVRKICQG IAFVGPAVCM IACAILTPAA   355
AtPHT4;4   LCVLPWLTMA VFANIGGWIA DTLVSRGL-S ITNVRKIMQS IGFLGPAF-- FLSQLSHVKT   447
AtPHT4;5   VSILPPLASI VVTSLASQFA DYLITNGV-D TTTVRKICQT IAFVAPAICM TLSSVDIGLP   417
AtPHT4;2   FSALPWATMA ISGYYAGAAS DFLIRTGH-S VTSVRKIMQS IGFMGPGL-- SLLCLNFAKS   418
AtPHT4;1   LSVFPWMTMA ISANAGGWIA DTLVSRGF-S VTNVRKIMQT IGFLGPAF-- FLTQLKHIDS   418
AtPHT4;6   SKMVPYLNMF VFSIVGGFIA DYLITKRILS VTRTRKFLNT VGFLIASAAL MVLPMFRTEN   329
AtPHT4;3   FSAVPWSMMA FTGYIAGFWS DLLIRRGT-S ITLTRKIMQS IGFIGPGI-- ALIGLTTAKQ   434

CrPHT4-7   AATAKAATTG VSAAGPVLTA VLVGLMSVAF ALGAWSRAGL YCNHQDLSPK YASALLGITN   415
AtPHT4;4   PAMA------ ---------- --VLCMACSQ GSDAFSQSGL YSNHQDIGPR YAGVLLGLSN   489
AtPHT4;5   PWEI------ ---------- --VGILTAGL ALSSFALSGL YCTHQDISPE YASILLGITN   458
AtPHT4;2   PSCA------ ---------- --AVFMTIAL SLSSFSQAGF LLNMQDIAPQ YAGFLHGISN   460
AtPHT4;1   PTMA------ ---------- --VLCMACSQ GTDAFSQSGL YSNHQDIAPR YSGVLLGLSN   460
AtPHT4;6   GV-------- ---------- --ILCSSVAL GFLALGRAGF AVNHMDIAPR YAGIVMGVSN   369
AtPHT4;3   PLVA------ ---------- --SAWLSLAV GLKSFSHLGF LINLQEIAPE YSGVLHGMCL   476

CrPHT4-7   TAGAIPGVLG VTMAGYLLDT T--------- -ASWANALFI PTAICQLFGA AVYTWLASSE   465
AtPHT4;4   TAGVLAGVFG TAATGYILQR ---------- -GSWD-DVFK VAVALYLIGT LVWNLFATGE   537
AtPHT4;5   TVGAVPGIVG VALTGFLLDS T--------- -HSWTMSLFV PSIFFYLTGT VVWLAFASSE   508
AtPHT4;2   CAGTLAAIVS TIGTGYFVQW L--------- -GSFQ-AFLT VTAFLYFATT VFWLLFATGE   509
AtPHT4;1   TAGVLAGVLG TAATGHILQH ---------- -GSWD-DVFT ISVGLYLVGT VIWNLFSTGE   508
AtPHT4;6   TAGTLAGIIG VDLTGKLLEA SKLVYSDLSH PESWRVVFFI PGLLC-IFSS VVFLLFSTGE   428
AtPHT4;3   TAGTLAAIVG TVGAGFFVEL L--------- -GSFQ-GFIL LTAILYLLSA LFYNIYATGE   525

CrPHT4-7   RQSWS----  470
AtPHT4;4   KILD-----  441
AtPHT4;5   PQTFRKEDS  517
AtPHT4;2   RVF------  512
AtPHT4;1   KIID-----  512
AtPHT4;6   RIFD-----  432
AtPHT4;3   RVDFDTTAA  534
```

Suppl. Figure 1. Amino acid sequence alignment of members of the PHT4 family in *Arabidopsis thaliana* (AtPHT4) and CrPHT4-7 in *C. reinhardtii*. Conserved amino acids are indicated in red. Predicted transmembrane regions are shown in green boxes. The amino acid sequence alignment and prediction of transmembrane helices were performed using the MultAlin and the Phyre2 v. 2.0 online software, respectively.

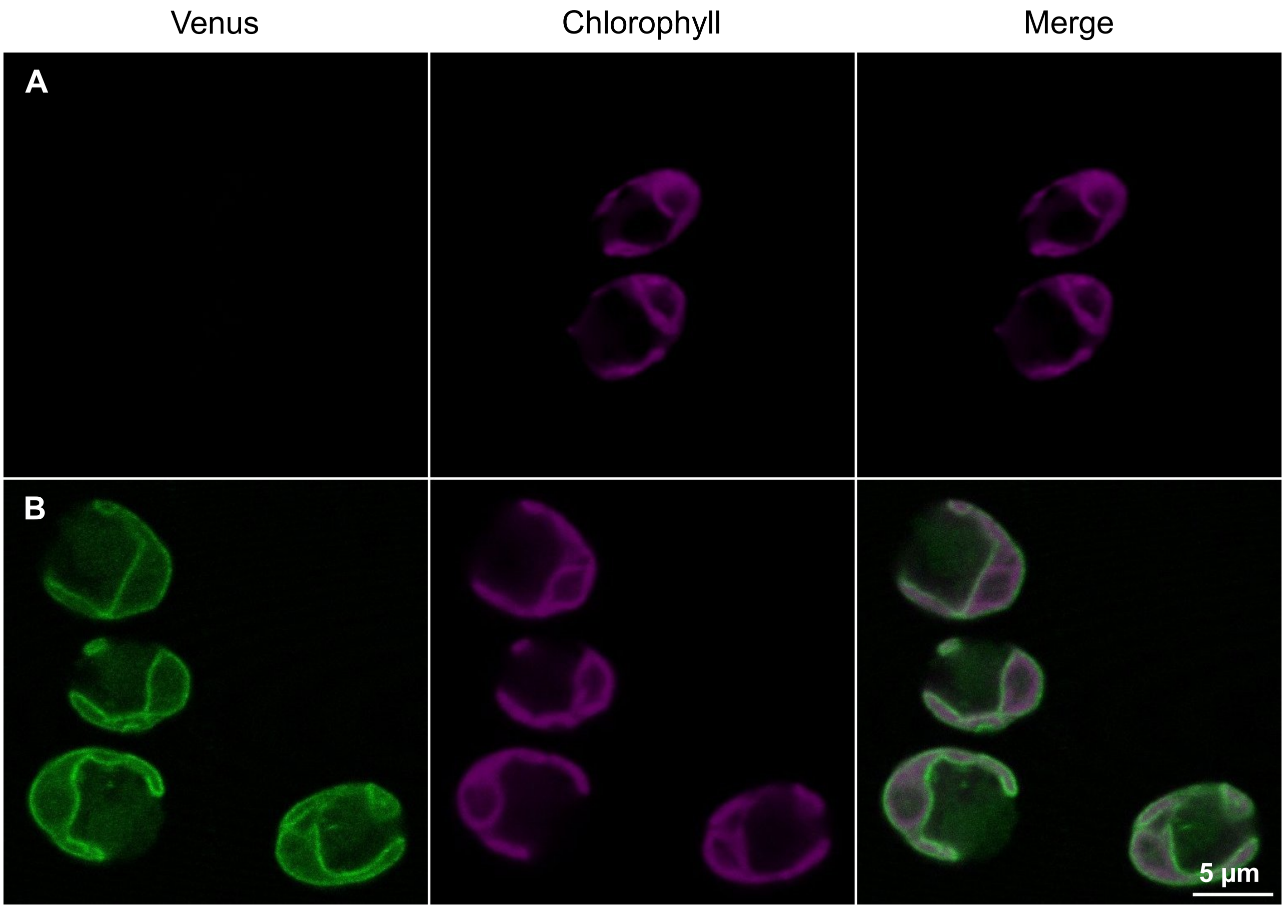

Supplemental Figure S2. Subcellular localization of CrPHT4-7 in the CC-4533 strain. A, Representative fluorescence microscopy images of CC-4533 and **B,** CC-4533 expressing pLM005-CrPHT4-7. Venus fluorescence and Chl autofluorescence were detected between 520-540 nm and 650-750 nm, respectively. The merged Venus + Chl autofluorescence image is also shown. Scale bar: 5 µm, applicable to all images.

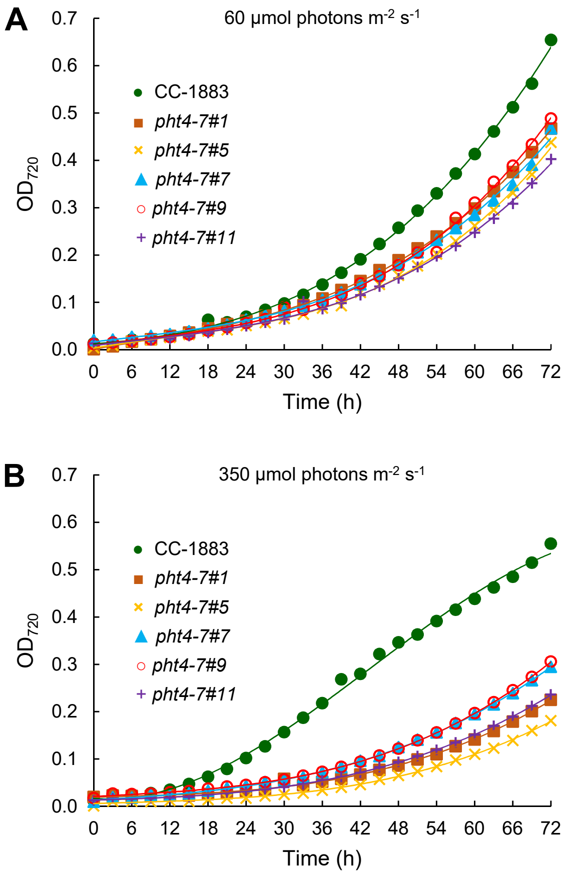

Supplemental Figure S3. Culture growth of independent *pht4-7* mutant lines generated by the CRISPR/Cas12a technique in TAP medium in continuous illumination in a Multi-Cultivator photobioreactor. A, Culture growth at 60 µmol photons m^{-2} s^{-1} as assessed by measuring optical density (OD) at 720 nm. **B,** Culture growth at 350 µmol photons m^{-2} s^{-1}. The initial Chl content was set to 0.5 µg Chl(a+b)/mL, the temperature was kept at 23°C, and the cultures were bubbled with air. The growth curves represent typical examples for each genotype.

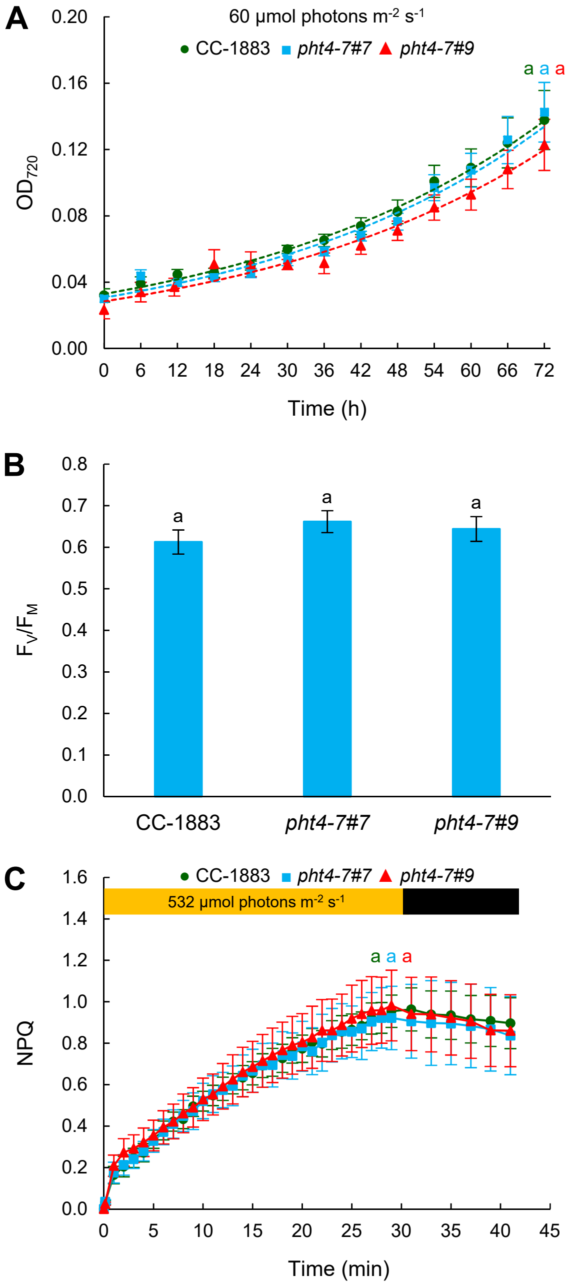

Supplemental Figure S4. Phenotypes of *pht4-7* mutants under photoautotrophic growth conditions. A, Culture growth of *pht4-7* mutants and the CC-1883 wild type, in HS medium in continuous illumination of 60 µmol photons m^{-2} s^{-1} at 23°C, bubbled with air for 72 h in a Multi-Cultivator photobioreactor. The initial Chl content was set to 0.5 µg Chl(a+b)/mL. **B,** F_V/F_M values after 72 h of growth in HS medium at 60 µmol photons m^{-2} s^{-1}. **C,** NPQ of cultures grown in HS medium at 60 µmol photons m^{-2} s^{-1}. The averages and standard errors are based on three independent experiments with one to two biological replicates in each. The significance of differences between means were determined by ANOVA with Tukey post-hoc test. The means with different letters are significantly different ($P < 0.05$). In the case of panel A significance was calculated for the last time point (72 h). In the case of panel C significance was calculated at the end of the illumination period.

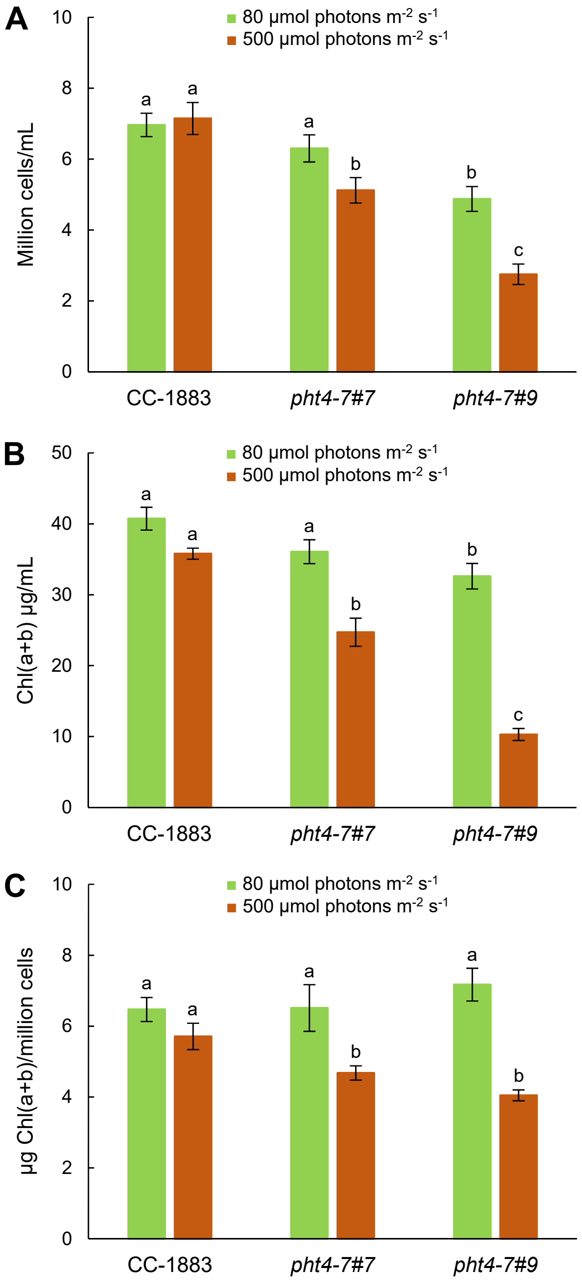

Supplemental Figure S5. Cell number and chlorophyll values of *pht4-7* mutants and the wild type grown in Erlenmeyer flasks. A, Cell numbers after 72 h of growth at 80 and 500 µmol photons m^{-2} s^{-1}. **B,** Chl(a+b) contents after 72 h of growth at 80 and 500 µmol photons m^{-2} s^{-1}. **C,** µg Chl(a+b)/million cells values after 72 h of growth at 80 and 500 µmol photons m^{-2} s^{-1}. The cultures were grown in Erlenmeyer flasks. The averages and standard errors are based on fifteen to twenty independent experiments with one to three biological replicates in each. The significance of differences between means were determined by ANOVA with Tukey post-hoc test. The means with different letters are significantly different ($P < 0.05$).

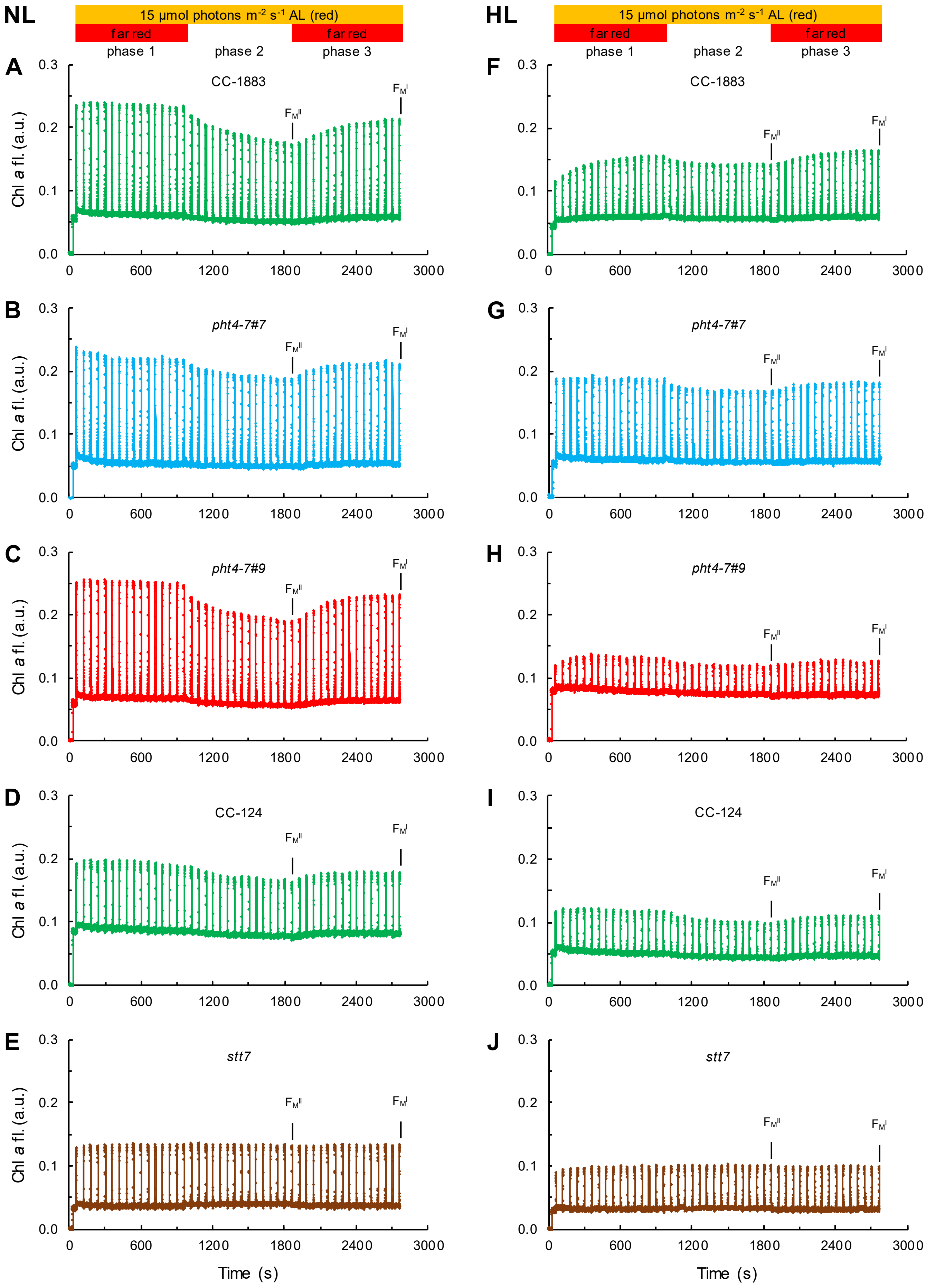

Supplemental Figure S6. Typical state transition kinetics of *pht4-7* and *stt7* mutants.. A-E, Cultures were grown in TAP medium in Erlenmeyer flasks under continuous illumination of 80 µmol photons $m^{-2}s^{-1}$. **F-J,** Cultures were grown in TAP medium under continuous illumination of 500 µmol photons $m^{-2}s^{-1}$. F_M^{II} and F_M^{I} values were used to calculate qT (see Materials and Methods)..

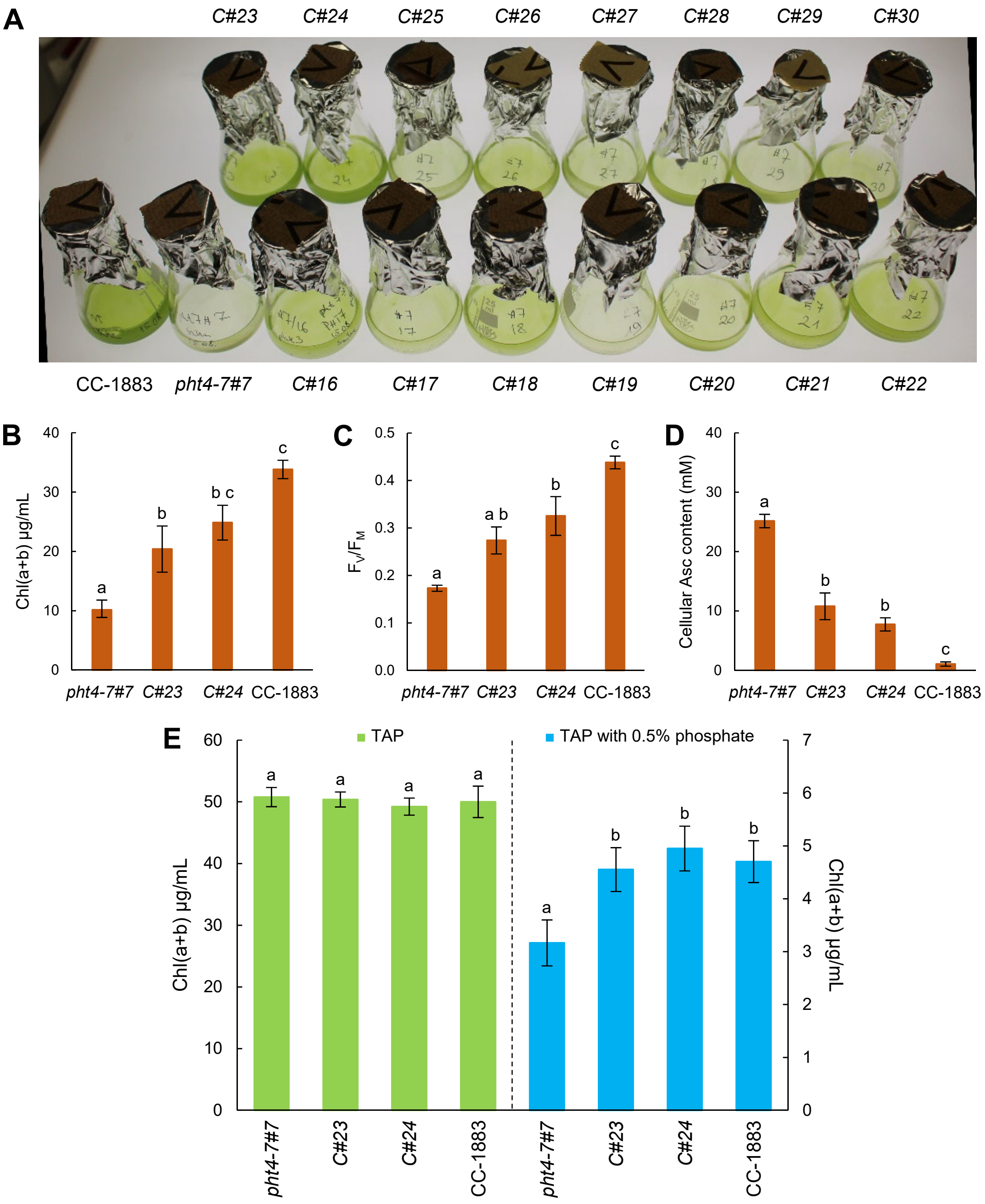

Supplemental Figure S7. Complementation of the *pht4-7#7* CRISPR/Cas12a mutant. A, Phenotype of the CC-1883 strain, the *pht4-7#7* mutant, and several randomly selected complementation lines grown for three days at 500 µmol photons m^{-2} s^{-1}. **B,** Chl(a+b) contents of the CC-1883 strain, the *pht4-7#7* mutant, and two selected complementation lines (*C#23*, *C#24*) at 500 µmol photons m^{-2} s^{-1}. **C,** F_V/F_M values under the same conditions. **D,** Ascorbate accumulation under the same conditions. **E,** Chl(a+b) contents after six days of phosphate deprivation at 80 µmol photons m^{-2} s^{-1}. The cultures were grown in Erlenmeyer flasks. The averages and error bars are based on four to ten independent experiments. The significance of differences between means were determined by ANOVA with Tukey post-hoc test. The means with different letters are significantly different ($P < 0.05$).
